# Volatile and non-volatile organic compounds synergistically determine quality in *Termitomyces eurhizus* from different regions

**DOI:** 10.1016/j.fochx.2026.104320

**Published:** 2026-08-13

**Authors:** Qian Zhang, Yanling Lu, Ying Chen, Fuyan Li, Jiaojiao Yang, Long Han, Qian Ma, Fangyu Fan

**Affiliations:** a College of Biological Science and Food Engineering, Southwest Forestry University, Kunming 650224, China; b Forest Resources Exploitation and Utilization Engineering Research Center for Grand Health of Yunnan Provincial Universities, Southwest Forestry University, Kunming 650224, China; c Key Laboratory of Forest Disaster Warning and Control of Yunnan Province, Southwest Forestry University, Kunming 650224, China

**Keywords:** *Termitomyces eurhizus*, Origins, Volatile and non-volatile compounds, HS-SPEM-GC–MS, Multivariate analysis

## Abstract

To investigate origin effects on *Termitomyces eurhizus*, this study evaluated volatile (VOCs) and non-volatile organic compounds (NVOCs) in samples from five regions and examined their correlations with environmental factors. Using headspace solid-phase microextraction coupled with gas chromatography–mass spectrometry (HS-SPME-GC–MS), electronic nose, high-performance liquid chromatography (HPLC), and ion chromatography, 91 VOCs were identified, among which were esters, aldehydes, ketones, and alcohols. The OPLS-DA model effectively distinguished samples from different regions, identifying esters and ketones as key differential markers and 1-octen-3-ol as the shared signature aroma. NVOCs analysis showed significant differences in protein, polysaccharides, total phenols, amino acids, and 5′-nucleotides among regions. The TAV values of 5′-GMP, 5′-AMP, glucose, and trehalose were all >1, making them major contributors to umami and sweetness. Further correlation analysis indicated that environmental factors significantly influenced metabolite accumulation. This study provides a scientific basis for origin traceability, quality evaluation, and targeted processing.

## Highlights

• Volatile and non-volatile compounds synergistically determine the quality of *Termitomyces eurhizus.*
• 91 VOCs identified; esters, aldehydes, ketones, and alcohols dominate.
• Lysine is the first limiting amino acid in *Termitomyces eurhizus*.
• 5′-GMP, 5′-AMP, glucose, and trehalose (TAV > 1) are key taste-active compounds.
• Environmental factors regulate compound accumulation, shaping origin-specific quality.

## Graphical abstract

**Figure:**
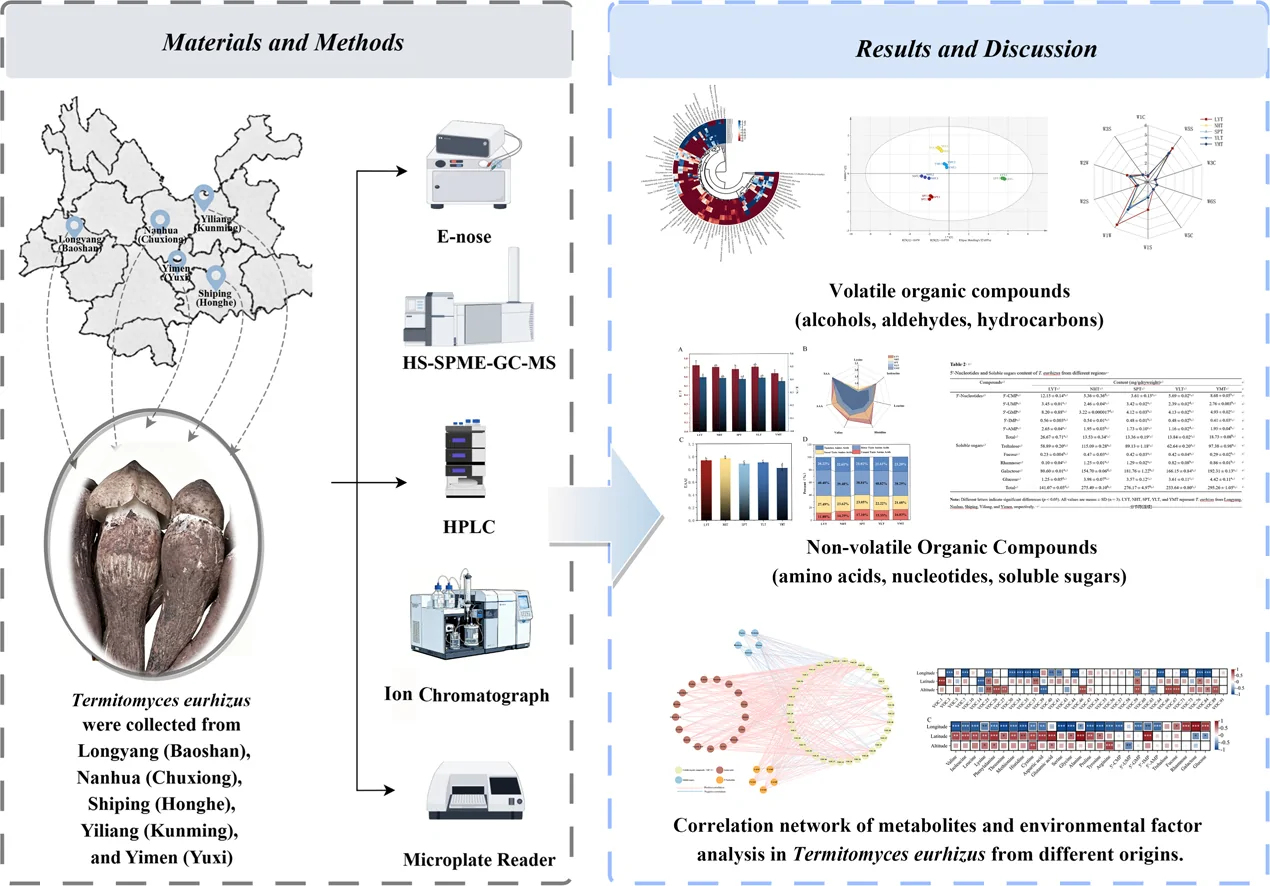

## 1 Introduction

Edible mushrooms are considered to possess nutritional, medicinal, and functional food potential due to their richness in high-quality proteins, dietary fiber, unsaturated fatty acids, and bioactive substances such as polysaccharides and sterols (Boro et al., 2025). However, the composition and content of these components are significantly influenced by species, substrate, and environmental factors, and the nutritional and chemical profiles of many wild mushroom species remain unstudied (Singh et al., 2025; Turfan et al., 2019). Therefore, conducting nutritional and phytochemical characterization of understudied wild edible mushrooms is a key step for assessing their food value and enabling sustainable exploitation. Moreover, organizations such as the Food and Agriculture Organization (FAO) recognize that the rational exploitation of wild edible mushrooms can enhance dietary nutrition, contribute to food security, support livelihoods, promote ecological conservation and add value to local economies (Saldanha et al., 2025). China is one of the leading nations in global wild edible mushroom production, with Yunnan Province in the southwest having become a major production center due to its unique natural conditions. According to statistics, 90% of the approximately 900 species of wild edible mushrooms in China are found in Yunnan. These wild edible mushrooms are a vital component of the local diet and an important source of income for local residents (Wang, Qu, et al., 2022).

*Termitomyces* is a highly representative wild edible mushroom in Yunnan. It forms a unique symbiotic relationship with termites and cannot be artificially cultivated, making it rare. This mushroom is characterized by its tender texture, unique flavor, and rich umami taste (Zhang et al., 2024). It is rich in protein, polysaccharides, and bioactive compounds (e.g., phenols and flavonoids) (Maity et al., 2020), and its nutritional value surpasses that of many other wild edible mushrooms (Gunasekara et al., 2021). Currently, research on *Termitomyces* has primarily focused on exploring artificial cultivation methods, as well as the identification and utilization of bioactive compounds. For instance, Yi et al. (2025) showed that arginine promotes the synthesis of cellulase by *Termitomyces*, thereby enhancing mycelial growth. Wu et al. (2025) found that methanol extracts of *Termitomyces* exert anti-obesity effects through multiple mechanisms, and that flavonoids serve as the key bioactive components. Notably, studies have confirmed that the origin of edible mushrooms has a significant impact on their quality (Xu et al., 2024). For instance, Zhou et al. (2025) found that environmental factors such as altitude, soil, and climate in different regions determine the quality of *Morchella*: samples from Gansu have higher glutamate content, resulting in a more prominent umami taste; samples from Henan have higher trehalose content, giving a sweeter taste; samples from Chongqing present an obvious sourness and grassy aroma; and samples from Yunnan are characterized by mushroom-like notes and fruity aromas. Li et al. (2024) also reported that differences in origin can influence the types, concentrations, and relative proportions of key volatile organic compounds (VOCs) in *Boletus* mushrooms, thereby contributing to their distinctive aromatic characteristics. Such origin-dependent differences may arise because regional environmental conditions, including climate, altitude, soil properties, and nutrient availability, can affect metabolic activity and thereby regulate the accumulation of flavor- and nutrition-related compounds in natural food materials (Li et al., 2020). However, although the effect of origin on the quality of wild edible mushrooms has been demonstrated, few reports have focused on *Termitomyces eurhizus* (*T. eurhizus*), a specific *Termitomyces* species that is widely consumed in Yunnan. Specifically, little research has been conducted on whether the flavor and nutritional properties of *T. eurhizus* vary with origin, and on how origin-specific environmental conditions shape its quality formation.

The quality characteristics of *T. eurhizus* can be analyzed from two aspects: VOCs and non-volatile organic compounds (NVOCs). For VOCs, headspace solid-phase microextraction coupled with gas chromatography–mass spectrometry (HS-SPM*E*-GC–MS) can be used to accurately identify VOCs (Xi et al., 2024), while an electronic nose (E-nose) can be used to rapidly characterize the overall flavor profile (Rong et al., 2023). These two approaches work in tandem to provide comprehensive analytical tools for studying the flavor of edible mushrooms, ranging from the microscopic to the macroscopic level. For example, Zhou et al. (2025) employed an E-nose and HS-SPME-GC–MS to analyze volatile compounds in morels, showing that the combined method effectively characterizes regional flavor differences in edible mushrooms. For NVOCs, high-performance liquid chromatography (HPLC) and ion chromatography are widely used for the determination of amino acids, soluble sugars, and 5′-nucleotides. In another study, HPLC-based analysis by Hu et al. (2020) revealed that the major NVOCs in the caps of *Stropharia rugosoannulata* were 5′-CMP, arabinose, malic acid, and succinic acid.

Accordingly, this study aims to analyze volatile and non-volatile organic compounds to compare the flavor profiles, taste characteristics, and nutritional properties of *T. eurhizus* from five different regions in Yunnan Province, thereby establishing a scientific foundation for origin traceability, quality evaluation, and targeted processing.

## 2 Materials and methods

### 2.1 *T. eurhizus*

**Fig. 1:**
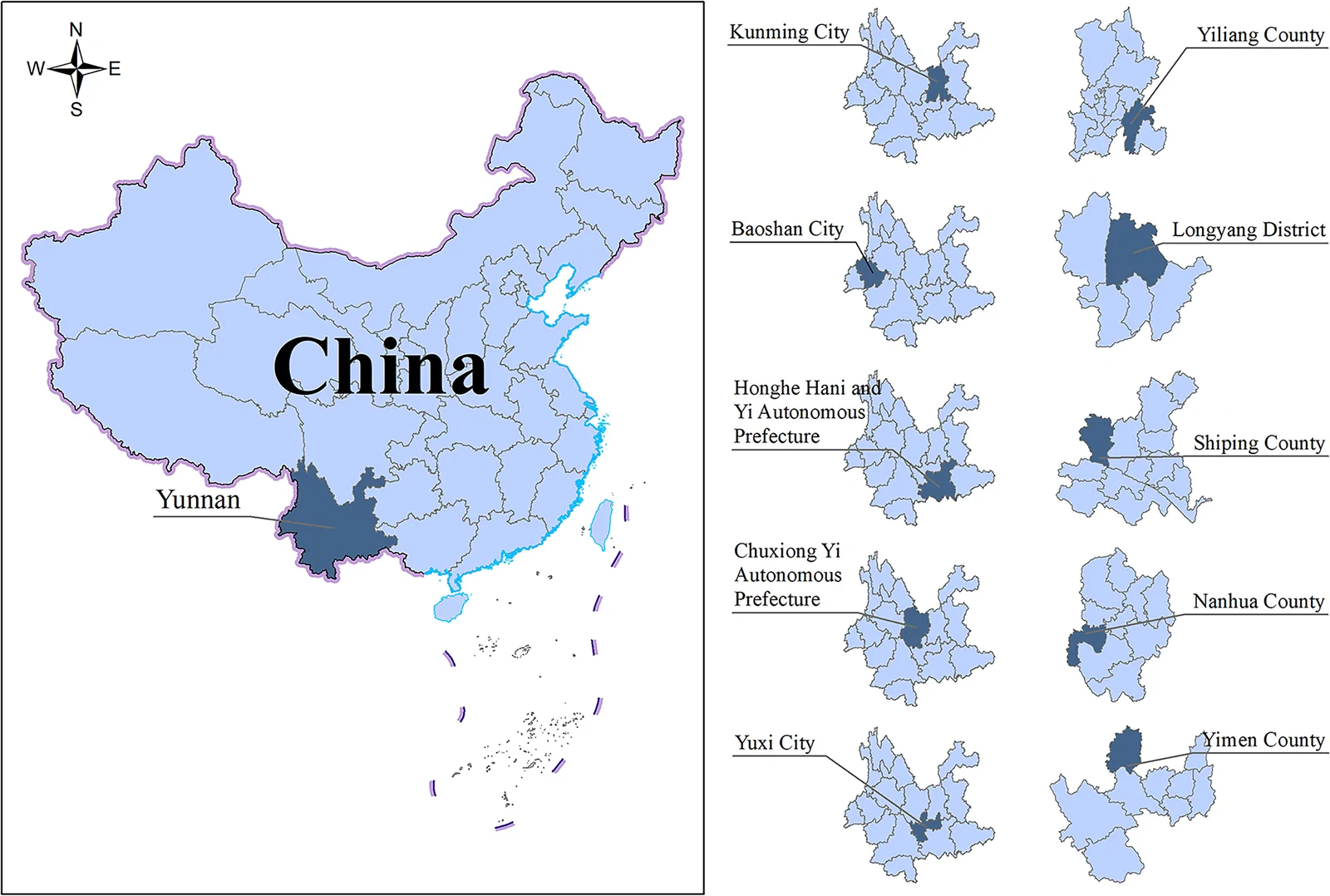
Geographic distribution of *T. eurhizus* sampling sites in Yunnan Province, China.

To confirm the authenticity of the sample origins, all materials were sourced directly from local farmers in each region. *T. eurhizus* samples with consistent freshness were collected from five locations in Yunnan Province, including Longyang, Baoshan; Nanhua, Chuxiong; Shiping, Honghe; Yiliang, Kunming; and Yimen, Yuxi. At each location, three independent biological replicates were collected. All samples were harvested in August 2025 during the peak fruiting season. Fig. 1 and Table S1 list the detailed collection locations for the five samples. All samples were identified as *T. eurhizus* through morphological analysis of characteristics, according to the taxonomic criteria of Pegler and Vanhaecke (1994). All fresh *T. eurhizus* were washed and subsequently freeze-dried using a YTLG-12A vacuum freeze dryer (Shanghai Yetuo Technology Co., Ltd., China). After drying, the samples were crushed, sieved with a 60-mesh sieve, and stored at −80 °C for future testing. The samples were labeled as follows: Longyang *T. eurhizus* (LYT), Nanhua *T. eurhizus* (NHT), Shiping *T. eurhizus* (SPT), Yiliang *T. eurhizus* (YLT), and Yimen *T. eurhizus* (YMT).

### 2.2 Chemicals

n-Hexane was purchased from Guangdong Guanghua Sci-Tech Co., Ltd. (Guangdong, China). 5′-Nucleotide standards (purity ≥99%), amino acid standards (purity ≥99%), and 1-decanol were obtained from Shanghai Yuanye Bio-Technology Co., Ltd. (Shanghai, China). All other chemical reagents were of analytical grade.

### 2.3 Electronic nose analysis

The PEN3 E-nose (Airsense Analytics GmbH, Schwerin, Germany) was applied to rapidly characterize the overall volatile profiles of the samples. The system consists of a headspace autosampler, 10 distinct metal oxide sensors arranged in an array, and an automated pattern recognition software. The 10 sensors and their primary target volatiles are detailed in Table S2. For analysis, a sample of 0.5 g was taken, placed into a headspace vial, capped, and equilibrated at room temperature for 30 min to allow for volatile compound enrichment. Subsequently, the headspace gas was injected into the E-nose for measurement. The headspace gas from each vial was sampled for a total duration of 100 s. The sampling was performed in a pulsed manner, with each injection lasting 1 s at a flow rate of 400 mL/min (Chen et al., 2020).

### 2.4 Headspace-solid phase microextraction-gas chromatography–mass spectrometry analysis

Extraction and analysis of VOCs followed the method of Li et al. (2024). A 0.5 g of sample was transferred into a headspace vial and spiked with 5.0 μL of 1-decanol (0.1 mg/mL). After equilibration for 28 min at 79 °C, a 50/30 μm DVB/CAR/PDMS fiber was inserted, and the sample was extracted for 23 min. Immediately thereafter, the fiber was inserted into the injection port at 250 °C and desorbed for 15 min. Subsequently, an HP-5MS column (30 m × 0.25 mm, 0.25 μm) was used for GC–MS (Shimadzu, Kyoto, Japan) analysis. The temperature program was as follows: 40 °C for 5 min, then ramp at 10 °C/min to 240 °C, hold 15 min. A flow rate of 1.0 mL/min was applied for the carrier gas, which consisted of helium with a purity of ≥99.999%. Based on the linear retention index method with n-alkanes as references, the experimental RI^a^ values of VOCs were obtained and compared with the corresponding theoretical RI^b^ values of certified standards from the NIST database (https://webbook.nist.gov/chemistry/). Eq. (1) was used to obtain the measured RI^a^ (Niu et al., 2022):

(1) $$\mathrm{RI}=100\times \left(n+\frac{T_{i}-T_{n}}{T_{n+1}-T_{n}}\right)$$

where *n* is the carbon number of the n-alkane, *T*_*i*_ is the retention time of the detected VOC in the sample, and *T*_*n*_ and *T*_*n+1*_ are the retention times of the adjacent n-alkanes before and after the target VOC, respectively (*T*_*n*_ < *T*_*i*_ < *T*_*n+1*_).

By employing the linear relationship of peak area to concentration for the internal standard (1-decanol, 0.1 mg/mL), a semi-quantitative technique was used to analyze VOCs. Eq. (2) was used to calculate the quantitative value (Yang et al., 2024):

(2) $$C=\frac{S_{1}\times m_{2}}{S_{2}\times m_{1}}$$

where *m*_*1*_ and *m*_*2*_ are the masses of the sample and the internal standard, respectively, and *S*_*1*_ and *S*_*2*_ are the peak areas of the target VOCs and the internal standard, respectively.

### 2.5 Nutritional content analysis

Kjeldahl analysis was performed to assess the protein content (Zhang et al., 2025), employing a nitrogen-to-protein conversion factor of 4.38 for mushrooms. Soxhlet extraction was performed to assess the fat content (Zheng et al., 2024). Following the assay of García et al. (2021) with minor adjustments, total phenolic levels were assessed using Folin-Ciocalteu reagent. The results were calculated from a gallic acid standard curve (0–100 mg/L) and expressed as mg of gallic acid equivalents per gram of dry sample (mg GAE/g DW). Polysaccharide content was determined using a phenol‑sulfuric acid protocol based on Yuan et al. (2022) with slight adjustments. The results were calculated from a d-glucose standard curve (0–60 mg/L) and expressed as mg of glucose equivalents per gram of dry weight (mg GE/g DW).

### 2.6 Amino acids content analysis

*T. eurhizus* were hydrolyzed with 6 M HCl at 105 °C for 22 h, followed by derivatization using phenyl isothiocyanate. The derivatized samples were extracted with n-hexane, filtered (0.22 μm), and subjected to HPLC analysis. Chromatographic separation was performed on a Shim-pack GIST C18 column (250 × 4.6 mm, 5 μm) at 40 °C using gradient elution, as described in previous study (Li et al., 2026).

Essential amino acid (EAA) scores were calculated using the FAO/WHO method, as shown in Eq. (3) (Hou et al., 2022):

(3) $$\begin{array}{l} \mathrm{AAS}=\frac{{\mathrm{AA}}_{\mathrm{test}}}{{\mathrm{AA}}_{\mathrm{ref}}} \end{array}$$

where *AA*_*test*_ is the content of a given amino acid in the test protein (mg/g protein), and *AA*_*ref*_ is the content of the corresponding amino acid in the FAO/WHO reference pattern (mg/g protein).

The essential amino acid index (EAAI) was calculated according to the following Eq. (4) (Sui & Harvey, 2021):

(4) EAAI=nlogEAA

In the formula, $\log\mathrm{EAA}=\left[\frac{1}{n}\right]\times \left[\log\left(\frac{100a_{1}}{a_{1}R}\right)+\ldots +\log\left(\frac{100a_{n}}{a_{n}R}\right)\right]$, *a*_*1*_ is the mass (mg) of a given amino acid in 1 g of the test protein, *a*_*1*_*R* is the mass (mg) of the corresponding amino acid in 1 g of the reference protein, and n is the number of amino acid species involved in the calculation.

### 2.7 5′-Nucleotides content analysis

A 0.5 g sample was mixed with 25 mL ultrapure water, ultrasonicated for 40 min, and then heated in a 100 °C water bath for 1 h. The mixture was cooled and centrifuged (8000 rpm, 10 min), and the supernatant was filtered through a 0.22 μm filter before analysis. Separation was performed on a C18 column (5 μm, 4.6 × 250 mm) using an isocratic mobile phase consisting of KH₂PO₄–K₂HPO₄ (0.02 M, pH 5.8, 1:1, v/v) and methanol (95:5, v/v). The column temperature, detection wavelength, injection volume, and flow rate were 35 °C, 254 nm, 10 μL, and 0.5 mL/min, respectively (Taylor et al., 1981).

### 2.8 Soluble sugars content analysis

As described by Zhou et al. (2025), a 0.5 g sample was mixed with 25 mL of ultrapure water and extracted in a water bath at 80 °C for 30 min. The extract was first centrifuged (8000 rpm, 10 min). After membrane filtration (0.22 μm) of the supernatant, the resulting sample was injected into an ICS-5000 ion chromatograph (Thermo Fisher Scientific, Waltham, USA) for analysis.

### 2.9 Taste activity values analysis

Taste activity values (TAV) are derived from the ratio of component concentration to its threshold. Only those with TAV > 1 are regarded as taste-active constituents in the food. TAV is calculated as Eq. (5) (Yin et al., 2022):

(5) $$\mathrm{TAV}=\frac{C}{T}$$

*C* denotes the actual content of the flavor compound (mg/g), and T represents its detection threshold (mg/g).

### 2.10 Statistical analysis

All data were obtained from at least three parallel experiments. Values are shown as means ± SD. Statistical analysis was conducted using one-way ANOVA in SPSS 27 (IBM, Armonk, NY, USA). Sampling maps were drawn using ArcGIS software. Origin 2021 software (OriginLab, Northampton, MA, USA) was employed to generate both the stacked bar charts and the radar charts. Cluster analysis heatmaps and correlation analysis heatmaps were produced via https://www.chiplot.online/. Multivariate analyses, specifically orthogonal partial least squares discriminant analysis (OPLS-DA), were conducted with SIMCA 14.1 (Umetrics, Malmo, Sweden). The correlation network was visualized using Cytoscape 3.10.3 (Cytoscape Consortium, San Diego, CA, USA).

## 3 Results and discussion

### 3.1 Volatile organic compounds characteristics of *T. eurhizus* from different regions

#### 3.1.1 Electronic nose analysis

**Fig. 2:**
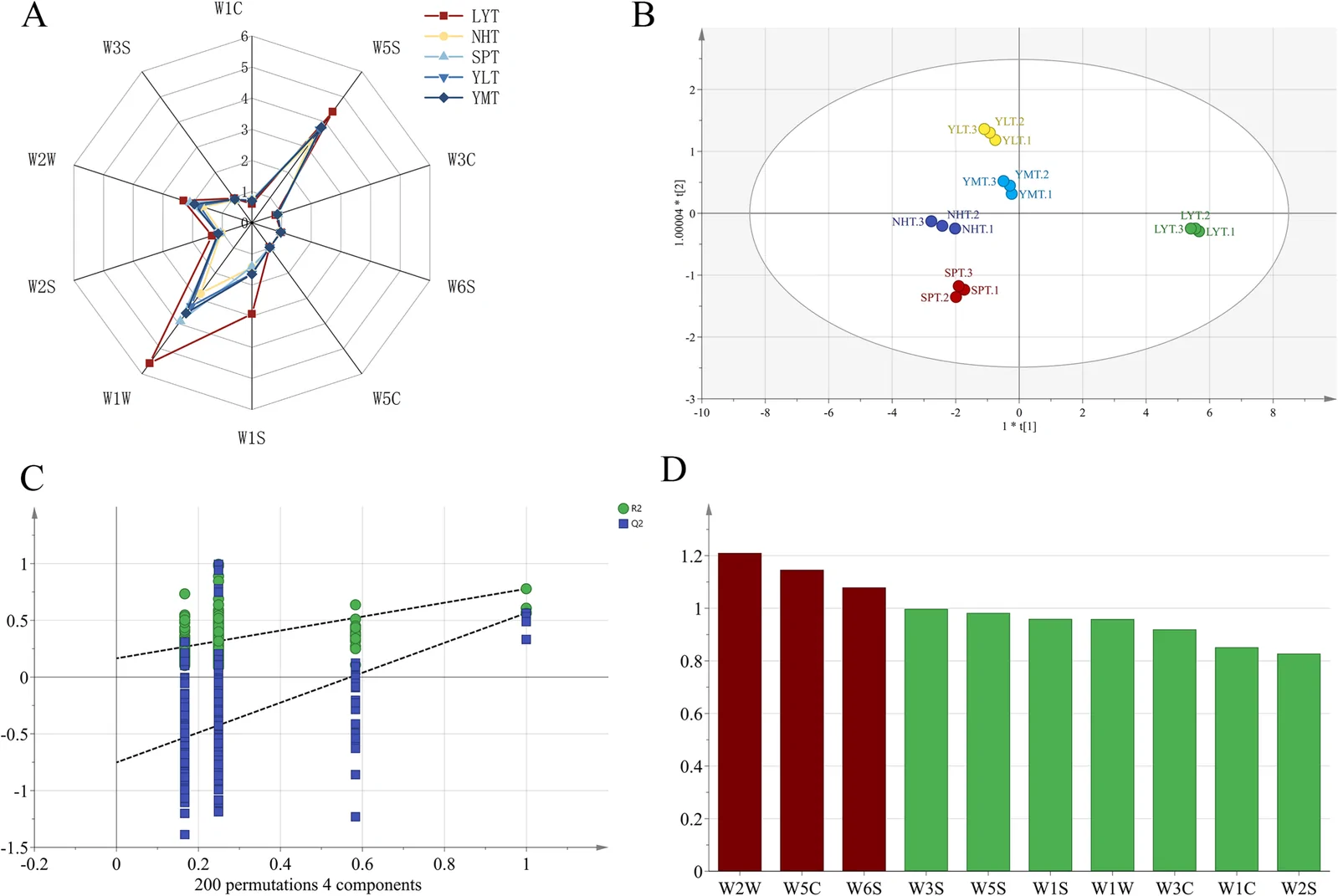
E-nose profiles and OPLS-DA analysis of *T. eurhizus* from different geographical origins. (A) Radar plot of sensor responses, (B) OPLS-DA score plot, (C) Results of permutation test (200 times), and (D) VIP scores. LYT, NHT, SPT, YLT, and YMT represent *T. eurhizus* from Longyang, Nanhua, Shiping, Yiliang, and Yimen, respectively.

E-nose systems emulate human sensory perception to capture the overall aromatic characteristics of samples, effectively avoiding the subjective inherent to organoleptic evaluation, and they identify variations in VOCs based on differences in sensor response values. Visual comparison of VOC signal intensities across origins is achieved by radar plots. Fig. 2A shows that the radar plots of samples from different origins have similar shapes, indicating that their VOCs exhibit relatively consistent overall response patterns on the sensors. Among them, sensors W1W and W5S are more sensitive to volatile components and exhibit higher response values, indicating that sulfur-containing compounds, nitrogen oxides, and terpenes in *T. eurhizus* significantly influence the aroma of the samples. At the same time, the W2S sensor used to detect alcohols did not exhibit a notable response. However, alcohols are crucial components that contribute to the characteristic aroma of edible mushrooms. This suggests that the E-nose does not fully capture the aroma compounds of *T. eurhizus* and can only characterize part of its flavor profile. This is in line with previous reports on Sichuan pepper (Feng et al., 2022) and *Boletus* mushrooms (Li et al., 2024).

Furthermore, an OPLS-DA model was established, and the results are presented in Fig. 2B. The explanatory variable coefficient of determination (R^2^X) for the OPLS-DA model was 0.998, the response variable coefficient of determination (R^2^Y) was 0.932, and the model prediction index (Q^2^) was 0.868. Both R^2^ and Q^2^ are greater than 0.5, suggesting that the model fits the data well (Feng et al., 2022). The model is considered reliable and is suggested to have a low risk of overfitting, as evidenced by the negative y-intercept of the Q^2^ regression line in Fig. 2C following 200 permutation tests. This model is suitable for the discriminant analysis of LYT, NHT, SPT, YLT, and YMT. In Fig. 2B, distinct separation between groups and tight clustering within groups can be observed, indicating good intra-group reproducibility. Additionally, Fig. 2D shows the variable importance in projection (VIP) generated by the OPLS-DA model. The VIP values can be employed to filter out variables that make a significant impact on classification, and variables with a VIP value >1 are typically regarded as key variables (Li et al., 2024). Among these, sensors W5C, W6S, and W2W all have VIP values >1, indicating that these three sensors contribute more significantly to sample classification and can serve as specific sensors for distinguishing *T. eurhizus* from different origins. Based on the above analysis, the E-nose can effectively distinguish between LYT, NHT, SPT, YLT, and YMT. However, the E-nose cannot provide specific information on the individual VOCs. Therefore, HS-SPME-GC–MS analysis was subsequently employed to characterize the VOCs in LYT, NHT, SPT, YLT, and YMT.

#### 3.1.2 Characterization of volatile organic compounds by headspace solid-phase microextraction-gas chromatography–mass spectrometry

**Fig. 3:**
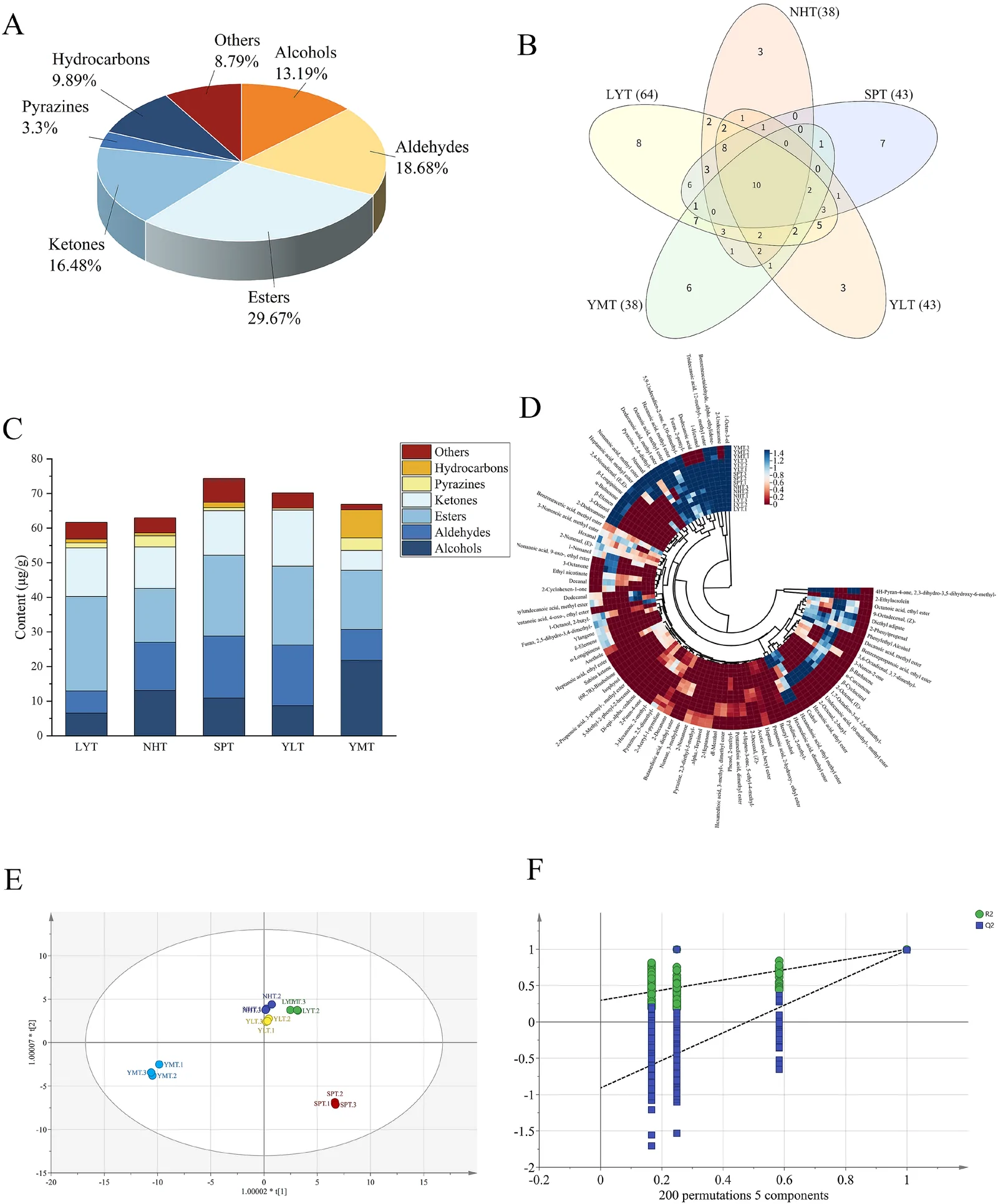
Analysis of VOCs in *T. eurhizus* from five origins. (A) Classification of 91 VOCs, (B) Venn diagram, (C) Classified bar chart of various VOCs content in different regions, (D) Heatmap of sample clustering, (E) OPLS-DA score plot, and (F) Results of permutation test (200 times). LYT, NHT, SPT, YLT, and YMT represent *T. eurhizus* from Longyang, Nanhua, Shiping, Yiliang, and Yimen, respectively.

HS-SPME-GC–MS analysis of LYT, NHT, SPT, YLT, and YMT led to the identification of 91 VOCs in total. As presented in Table 1 and Fig. 3A, these compounds can be classified into seven major categories, including 12 alcohols (13.19%), 17 aldehydes (18.68%), 27 esters (29.67%), 15 ketones (16.48%), 3 pyrazines (3.30%), 9 hydrocarbons (9.89%), and 8 other compounds (8.79%). Among these, esters comprised the largest proportion, with aldehydes and ketones ranking next, while alcohols (e.g., 1-octen-3-ol) are characteristic volatile compounds commonly reported in edible mushrooms (Li et al., 2024; Liu et al., 2021). These four categories together accounted for 78.02% of the total, constituting the main components of the volatile constituents in *T. eurhizus*. Venn diagram analysis (Fig. 3B) shows that the five origins collectively contain 10 types of VOCs, including mushroom aroma (1-octen-3-ol), grassy aroma ((E)-2-nonenal), fruity notes (Methyl hexanoate), sweet and floral notes (Methyl phenylacetate), roasted nut notes (2,6-Diethylpyrazine), and fatty, wax (Methyl caprylate), collectively forming the fundamental aromatic framework of *T. eurhizus*.

**Table 1:** Volatile organic compounds in *T. eurhizus* from different origins.

No.	CAS	Compounds	Formula	Content (μg/g)					RT	RI^a^	RI^b^	Identification Method	Odor description	VIP
				LYT	NHT	SPT	YLT	YMT						
**Alcohols**														
VOC.1	111-27-3	1-Hexanol	C_6_H_14_O	1.58 ± 0.17^c^	3.28 ± 0.30^a^	2.34±0.32^b^	2.14 ± 0.10^b^	-	7.55	860	860	MS/RI	ethereal, fusel oil, fruity, alcoholic, sweet, green	1.00424
VOC.2	3391-86-4	1-Octen-3-ol	C_8_H_16_O	1.82 ± 0.15^c^	6.52 ± 0.69^b^	2.96±0.07^c^	3.47 ± 0.35^c^	18.76 ± 2.37^a^	10.78	969	963	MS/RI	mushroom, earthy, green, oily, fungal, raw chicken	0.94107
VOC.3	100-51-6	Benzyl alcohol	C_7_H_8_O	0.03 ± 0.02^a^	-	-	-	-	12.18	1036	1028	MS/RI	floral, rose, phenolic, balsamic	1.00005
VOC.4	60-12-8	Phenylethyl Alcohol	C_8_H_10_O	1.11 ± 0.02^b^	1.26 ± 0.09^a^	0.63±0.06^d^	0.90 ± 0.05^c^	-	13.99	1136	1128	MS/RI	N	0.99281
VOC.5	143-08-8	1-Nonanol	C_9_H_20_O	-	0.78 ± 0.02^b^	-	1.36 ± 0.01^a^	0.75 ± 0.18^b^	15.25	1159	1157	MS/RI	fresh, clean, fatty, floral, rose, orange, dusty, wet, oily	1.02689
VOC.6	3913-02-8	2-Butyl-1-octanol	C_12_H_26_O	-	0.23 ± 0.02^c^	-	0.49 ± 0.13^b^	1.02 ± 0.15^a^	17.09	1293	1277	MS/RI	N	0.917607
VOC.7	505-32-8	Isophytol	C_20_H_40_O	0.65 ± 0.03^a^	-	-	-	-	19.78	1899	1922	MS/RI	mild, floral, herbal, green	1.08158
VOC.8	77-53-2	Cedrol	C_15_H_26_O	1.34 ± 0.19^b^	0.99 ± 0.15^c^	3.40±0.10^a^	-	-	23.31	1543	1562	MS/RI	cedarwood, woody, dry, sweet, soft	0.97847
VOC.9	22460-59-9	2,6-Dimethyl-1,7-octadien-3-ol	C_10_H_18_O	-	-	1.59±0.06^a^	-	-	19.77	1071	1095	MS/RI	N	0.99885
VOC.10	89-78-1	Menthol	C_10_H_20_O	-	-	-	0.28 ± 0.01^a^	-	15.43	1164	1166	MS/RI	peppermint, cool, woody	1.09208
VOC.11	589-98-0	3-Octanol	C_8_H_18_O	-	-	-	-	2.43 ± 0.67^a^	11.23	979	985	MS/RI	earthy, mushroom, herbal, melon, citrus, woody, spicy, minty	0.91886
VOC.12	98-55-5	α-Terpineol	C_10_H_18_O	-	-	-	-	0.27 ± 0.06^a^	15.78	1143	1144	MS/RI	pine, terpene, lilac, citrus, woody, floral	0.93653
**Aldehydes**														
VOC.13	66-25-1	Hexanal	C_6_H_12_O	0.30 ± 0.07^b^	0.46 ± 0.12^ab^	0.78±0.33^ab^	0.49 ± 0.19^ab^	0.88 ± 0.35^a^	5.25	806	801	MS/RI	fresh, green, fatty, aldehydic, grass, leafy, fruity, sweaty	0.77104
VOC.14	111-71-7	Heptanal	C_7_H_14_O	0.10 ± 0.01^b^	-	0.08±0.01^b^	0.05 ± 0.02^c^	0.19 ± 0.01^a^	8.53	905	902	MS/RI	fresh, aldehydic, fatty, green, herbal, wine-lee, ozone	1.01909
VOC.15	2548-87-0	(E)-2-Octenal	C_8_H_14_O	-	-	1.29±0.20^a^	-	-	12.71	1023	1030	MS/RI	fresh, cucumber, fatty, green, herbal, banana, waxy, green leaf	0.98844
VOC.16	4432-63-7	2-Phenylpropenal	C_9_H_8_O	1.04 ± 0.01^b^	1.48 ± 0.27^a^	1.11±0.33^a^	1.15 ± 0.05^a^	-	14.91	1148	1150	MS/RI	N	0.95087
VOC.17	18829-56-6	(E)-2-Nonenal	C_9_H_16_O	0.60 ± 0.01^c^	0.57 ± 0.05^c^	0.64±0.02^bc^	0.74 ± 0.04^b^	0.96 ± 0.16^a^	15.00	1112	1132	MS/RI	fatty, green, cucumber, aldehydic, citrus	0.91319
VOC.18	112-31-2	Decanal	C_10_H_20_O	-	-	0.37±0.05^b^	1.05 ± 0.02^a^	-	15.96	1204	1207	MS/RI	sweet, aldehydic, waxy, orange peel, citrus, floral	0.97610
VOC.19	2497-25-8	(Z)-2-Decenal	C_10_H_18_O	-	-	0.22±0.01^a^	-	-	16.04	1212	1227	MS/RI	tallow	0.99891
VOC.20	432-25-7	β-Cyclocitral	C_10_H_16_O	-	-	1.18±0.16^a^	-	-	16.71	1204	1196	MS/RI	tropical, saffron, herbal, clean, rose oxide, sweet, tobacco, damascone, fruity	0.99256
VOC.21	4411-89-6	2-Phenyl-2-butenal	C_10_H_10_O	3.00 ± 0.29^c^	4.70 ± 1.29^b^	6.56±0.99^a^	2.98 ± 0.18^c^	1.82 ± 0.52^c^	17.24	1265	1273	MS/RI	sweet, narcissus, cortex, beany, honey, cocoa, nutty, radish	0.95769
VOC.22	13019-16-4	2-Butyl-2-octenal	C_12_H_22_O	-	-	2.58±0.16^a^	-	-	17.92	1388	1389	MS/RI	aldehydic, green, watery, fruity, metallic, oily, tropical, fatty, sweaty, goaty	0.99825
VOC.23	2423-10-1	(Z)-9-Octadecenal	C_18_H_34_O	0.71 ± 0.18^b^	0.38 ± 0.10^c^	1.64±0.05^a^	0.85 ± 0.13^b^	-	20.27	2007	1995	MS/RI	N	0.94999
VOC.24	55722-59-3	3,7-Dimethyl-3,6-octadienal	C_10_H_16_O	0.47 ± 0.04^b^	-	1.42±0.23^a^	-	-	16.80	1174	1183.9	MS/RI	N	0.97568
VOC.25	21834-92-4	5-Methyl-2-phenyl-2-hexenal	C_13_H_16_O	0.19 ± 0.03^a^	0.20 ± 0.07^a^	-	-	-	21.12	1499	1486	MS/RI	N	1.02622
VOC.26	922-63-4	2-Ethylacrolein	C_5_H_8_O	-	4.84 ± 1.00^a^	-	-	-	3.63	674		MS/RI	N	1.14355
VOC.27	112-54-9	Dodecanal	C_12_H_24_O	-	1.28 ± 0.33^a^	-	-	0.66 ± 0.04^b^	15.97	1402	1412	MS/RI	soapy, waxy, aldehydic, citrus, green, floral	1.04407
VOC.28	124-19-6	Nonanal	C_9_H_18_O	-	-	-	1.62 ± 0.12^b^	2.80 ± 0.12^a^	13.80	1104	1102	MS/RI	waxy, aldehydic, rose, fresh, orris, orange peel, fatty, peely	0.96739
VOC.29	5910-87-2	(E, E)-2,4-Nonadienal	C_9_H_14_O	-	-	-	-	1.57 ± 0.17^a^	11.01	1210	1207	MS/RI	fatty, melon, waxy, green, violet leaf, cucumber, tropical fruit, chicken fat	0.93959

**Esters**														
VOC.30	97-64-3	Ethyl lactate	C_5_H_10_O_3_	0.14 ± 0.04^a^	-	-	-	-	5.63	848	836	MS/RI	sharp, tart, fruity, buttery, butterscotch	1.04612
VOC.31	106-70-7	Methyl hexanoate	C_7_H_14_O_2_	1.01 ± 0.08^c^	1.24 ± 0.25^bc^	1.92±0.50^b^	0.96 ± 0.20^c^	2.07 ± 0.71^a^	9.21	884	902	MS/RI	ethereal, fruity, pineapple, apricot, strawberry, tropical fruit, banana, bacon	0.85803
VOC.32	123-66-0	Ethyl hexanoate	C_8_H_16_O_2_	1.32 ± 0.09^b^	-	2.30±0.38^a^	-	-	11.26	984	982	MS/RI	sweet, fruity, pineapple, waxy, green, banana	0.97925
VOC.33	106-73-0	Methyl heptanoate	C_8_H_16_O_2_	1.05 ± 0.08b	-	-	0.66 ± 0.06b	1.89 ± 0.38a	11.88	984	1004	MS/RI	N	0.99366
VOC.34	539-88-8	Ethyl 4-oxovalerate	C_7_H_12_O_3_	1.35 ± 0.09a	-	-	-	-	12.75	1020	1024	MS/RI	sweet, fruity, floral, berry, green, pineapple, rhubarb	1.08062
VOC.35	106-30-9	Ethyl heptanoate	C_9_H_18_O_2_	0.71 ± 0.07a	-	-	-	-	13.62	1083	1081	MS/RI	fruity, pineapple, cognac, rum, wine	1.07635
VOC.36	111-11-5	Methyl caprylate	C_9_H_18_O_2_	1.99 ± 0.20^a^	1.86 ± 0.03^a^	1.37±0.21^b^	1.92 ± 0.05^a^	2.17 ± 0.40^a^	14.21	1083	1107	MS/RI	waxy, green, sweet, orange, aldehydic, vegetable, herbal	0.85711
VOC.37	101-41-7	Methyl phenylacetate	C_9_H_10_O_2_	1.11 ± 0.08^a^	0.74 ± 0.11^b^	0.45±0.07^c^	0.67 ± 0.03^b^	0.37 ± 0.03^c^	15.32	1160	1156	MS/RI	sweet, floral, honey, spice, waxy, almond	1.00668
VOC.38	106-32-1	Ethyl octanoate	C_10_H_20_O_2_	1.02 ± 0.04^b^	0.60 ± 0.17^c^	1.52±0.08^a^	0.91 ± 0.12^b^	-	15.75	1183	1187	MS/RI	fruity, wine, waxy, sweet, apricot, banana, brandy, pear	0.92711
VOC.39	13481-87-3	Methyl 3-nonenoate	C_10_H_18_O_2_	0.56 ± 0.03^b^	-	0.96±0.13^a^	1.01 ± 0.08^a^	0.69 ± 0.14^b^	16.22	1191		MS/RI	fruity, green, watermelon rind, pear skin, honeydew, juicy, fatty, dry	1.09926
VOC.40	627-93-0	Dimethyl adipate	C_8_H_14_O_4_	3.72 ± 1.05^a^	-	0.56±0.08^c^	2.04 ± 0.12^b^	-	16.64	1195	1206	MS/RI	mild, nutty	1.07086
VOC.41	18891-13-9	Ethyl methyl adipate	C_9_H_16_O_4_	3.33 ± 0.38^a^	0.34 ± 0.01^d^	0.96±0.02^c^	2.03 ± 0.14^b^	-	18.00	1250		MS/RI	N	1.08184
VOC.42	2021-28-5	Ethyl 3-phenylpropanoate	C_11_H_14_O_2_	0.77 ± 0.09^b^	-	1.53±0.16^a^	-	-	18.70	1359	1355	MS/RI	hyacinth, rose, honey, fruity, rum	0.98325
VOC.43	141-28-6	Diethyl adipate	C_10_H_18_O_4_	1.00 ± 0.14^c^	-	1.12±0.22^bc^	1.42 ± 0.07^a^	-	19.28	1350	1349	MS/RI	N	1.05542
VOC.44	103-26-4	Methyl cinnamate	C_10_H_10_O_2_	0.62 ± 0.04^a^	-	-	-	-	19.39	1367	1377	MS/RI	sweet, balsam, strawberry, cherry, cinnamon	1.08023
VOC.45	111-82-0	Methyl dodecanoate	C_13_H_26_O_2_	1.56 ± 0.37^b^	1.76 ± 0.44^b^	-	2.18 ± 0.27^ab^	2.79 ± 0.54^a^	21.71	1481	1507	MS/RI	waxy, soapy, creamy, coconut, mushroom	0.91128
VOC.46	29811-50-5	Octyl 2-methylbutyrate	C_13_H_26_O_2_	0.29 ± 0.06^c^	1.49 ± 0.18^a^	-	-	0.78 ± 0.21^c^	22.32	1417		MS/RI	N	1.10717
VOC.47	614-18-6	Ethyl nicotinate	C_8_H_9_NO_2_	-	0.58 ± 0.06^b^	0.37±0.03^c^	0.76 ± 0.02^a^	-	16.14	1154	1771	MS/RI	N	1.05842
VOC.48	110-42-9	Methyl decanoate	C_11_H_22_O_2_	2.08 ± 0.13^a^	0.96 ± 0.06^b^	1.39±0.37^b^	-	-	16.30	1282	1306	MS/RI	oily, wine, fruity, floral	0.98847
VOC.49	5129-58-8	Methyl 12-methyltridecanoate	C_15_H_30_O_2_	3.69 ± 0.88^c^	6.01 ± 0.51^a^	5.29±0.07^ab^	4.76 ± 0.24b^c^	4.50 ± 0.75^bc^	24.83	1675	1678	MS/RI	N	0.98992
VOC.50	142-92-7	Acetic acid, hexyl ester	C_8_H_16_O_2_	-	-	0.25±0.09^a^	-	-	11.60	984	993	MS/RI	fruity, green, apple, banana, sweet	0.94859
VOC.51	123-25-1	Hexyl acetate	C_8_H_14_O_4_	-	-	0.41±0.02^a^	-	-	15.36	1151	1149	MS/RI	mild, fruity, cooked apple, ylang	0.99876
VOC.52	1731-84-6	Methyl nonanoate	C_10_H_20_O_2_	-	-	-	1.64 ± 0.24^a^	1.88 ± 0.38^a^	16.29	1183	1205	MS/RI	sweet, fruity, pear, waxy, tropical, wine	0.99093
VOC.53	5129-56-6	Methyl 10-methylundecanoate	C_13_H_26_O_2_	-	-	3.00±0.11^a^	-	-	21.70	1417		MS/RI	N	0.99902
VOC.54	1119-40-0	Dimethyl glutarate	C_7_H_12_O_4_	-	-	-	0.10 ± 0.01^a^	-	14.44	1122	1135	MS/RI	estery, floral	1.09107
VOC.55	54576-13-5	Dimethyl 3-methylhexanedioate	C_9_H_16_O_4_	-	-	-	0.36 ± 0.02^a^	-	17.46	1186	1253	MS/RI	N	1.09218
VOC.56	3433-16-7	Ethyl 9-oxononanoate	C_11_H_20_O_3_	-	-	-	1.32 ± 0.02^a^	-	19.51	1470	1502	MS/RI	N	1.09198

**Ketones**														
VOC.57	930-68-7	2-Cyclohexen-1-one	C_6_H_8_O	-	-	-	0.75 ± 0.15^a^	-	10.66	873	885	MS/RI	roasted, savory, green	1.06987
VOC.58	106-68-3	3-Octanone	C_8_H_16_O	-	-	-	1.42 ± 0.15^a^	-	12.75	952	965	MS/RI	fresh, herbal, lavender, sweet, mushroom	1.08858
VOC.59	28564-83-2	3,5-Dihydroxy-6-methyl-2,3-dihydro-4H-pyran-4-one	C_6_H_8_O_4_	4.92 ± 0.14^a^	4.81 ± 0.98^a^	-	4.25 ± 0.39^a^	-	14.71	1169	1162	MS/RI	N	1.01527
VOC.60	22319-28-4	5-ethyl-4-methyl-4-hepten-3-one	C_10_H_18_O	-	-	-	0.19 ± 0.03^b^	-	16.40	1113		MS/RI	N	1.08161
VOC.61	112-12-9	2-Undecanone	C_11_H_22_O	5.79 ± 0.33^c^	4.49 ± 0.50^d^	8.85±0.05^a^	6.46 ± 0.33^b^	-	17.67	1251	1256	MS/RI	waxy, fruity, creamy, fatty, orris, floral	0.93594
VOC.62	689-67-8	6,10-Dimethyl-5,9-undecadien-2-one	C_13_H_22_O	1.02 ± 0.12^b^	1.37 ± 0.39^b^	2.51±0.04^a^	2.55 ± 0.28^a^	2.20 ± 0.62^a^	20.46	1420	1433	MS/RI	fresh, rose leaf, floral, green, magnolia, aldehydic, fruity	0.99157
VOC.63	693-54-9	2-Decanone	C_10_H_20_O	0.36 ± 0.00^a^	-	0.37±0.05^a^	-	0.26 ± 0.06^b^	15.66	1151	1154	MS/RI	orange, floral, fatty, peach	1.05642
VOC.64	513-20-2	Sabina ketone	C_9_H_14_O	0.68 ± 0.01^a^	-	-	-	-	16.15	1146	1156	MS/RI	N	1.08197
VOC.65	14309-57-0	3-Nonen-2-one	C_9_H_16_O	0.64 ± 0.05^a^	-	1.14±0.07^b^	0.63 ± 0.02^c^	-	18.54	1137	1142	MS/RI	fruity, berry, fatty, oily, ketonic, weedy, spicy, licorice	0.99747
VOC.66	1196-01-6	2-Pinen-4-one	C_10_H_14_O	-	0.50 ± 0.07^a^	-	-	-	16.05	1190	1204	MS/RI	spicy, mint, camphor	1.13505
VOC.67	7379-12-6	2-Methyl-3-hexanone	C_7_H_14_O	-	0.83 ± 0.03^a^	-	-	-	22.79	789	784	MS/RI	fruity	1.14706
VOC.68	110-43-0	2-Heptanone	C_7_H_14_O	-	-	0.75±0.10^a^	-	0.29 ± 0.03^b^	8.15	853	868	MS/RI	fruity, spicy, sweet, herbal, coconut, woody	0.94530
VOC.69	821-55-6	2-Nonanone	C_9_H_18_O	-	-	-	-	0.38 ± 0.07^a^	13.47	1052	1059	MS/RI	fresh, sweet, green, weedy, earthy, herbal	0.94152
VOC.70	6175-49-1	2-Dodecanone	C_12_H_24_O	-	-	-	-	2.60 ± 0.54^a^	17.65	1350	1348	MS/RI	fresh, sweet, green, weedy, earthy, herbal	0.93833
VOC.71	72441-71-5	(6R,7R)-Bisabolone	C_15_H_24_O	0.61 ± 0.12^a^	-	-	-	-	25.21	1740	1747	MS/RI	N	1.00997

**Pyrazines**														
VOC.72	123-32-0	2,5-Dimethylpyrazine	C_6_H_8_N_2_	0.14 ± 0.04^b^	0.47 ± 0.10^a^	-	-	0.48 ± 0.36^a^	8.80	894	890	MS/RI	musty, potato, cocoa, nutty, fatty, oily, nuance	0.89723
VOC.73	13067-27-1	2,6-Diethylpyrazine	C_8_H_12_N_2_	1.32 ± 0.07^b^	2.75 ± 0.31^a^	0.89±0.29^c^	0.53 ± 0.01^c^	2.88 ± 0.25^a^	13.13	1093	1089	MS/RI	nutty, hazelnut	1.03975
VOC.74	18138-04-0	2,3-Diethyl-6-methylpyrazine	C_9_H_14_N_2_	-	-	-	-	0.24 ± 0.03^a^	14.81	1206	1171	MS/RI	musty, nutty, meaty, vegetable, roasted, hazelnut	0.94452

**Hydrocarbons**														
VOC.75	51655-64-2	2-Ethyl-1-octene	C_10_H_20_	-	-	0.57±0.04^a^	-	-	19.36	982	987	MS/RI	N	0.99731
VOC.76	50894-66-1	Di-epi-α-cedrene	C_15_H_24_	0.49 ± 0.02^a^	0.30 ± 0.16^b^	-	-	-	20.09	1403	1398	MS/RI	N	1.00992
VOC.77	72346-55-5	β-Barbatene	C_15_H_24_	0.59 ± 0.01^b^	0.61 ± 0.23^b^	0.93±0.03^a^	-	-	20.69	1421	1440	MS/RI	N	0.98131
VOC.78	20307-84-0	δ-Elemene	C_15_H_24_	-	-	-	-	0.88 ± 0.14^a^	18.47	1377	1386	MS/RI	sweet, herbal, woody	0.94340
VOC.79	5989-08-2	α-Longipinene	C_15_H_24_	-	-	-	-	0.94 ± 0.14^a^	18.88	1403	1395	MS/RI	N	0.94386
VOC.80	3691-11-0	α-Bulnesene	C_15_H_24_	-	-	-	-	1.66 ± 0.27^a^	19.31	1490	1490	MS/RI	N	0.94291
VOC.81	41432-70-6	β-Longipinene	C_15_H_24_	-	-	-	-	2.01 ± 0.46^a^	19.80	1398	1402	MS/RI	N	0.93032
VOC.82	14912-44-8	Ylangene	C_15_H_24_	-	-	-	-	0.80 ± 0.21^a^	20.08	1369	1370	MS/RI	N	0.93466
VOC.83	515-13-9	β-Elemen	C_15_H_24_	-	-	-	-	1.85 ± 0.43^a^	21.54	1398	1389	MS/RI	sweet	0.93588

**Other Compounds**														
VOC.84	4180-23-8	Anethole	C_10_H_12_O	0.80 ± 0.05^a^	0.53 ± 0.14^b^	-	-	0.23 ± 0.03^c^	17.58	1290	1283	MS/RI	sweet, anise, licorice, mimosa	1.04409
VOC.85	53720-72-2	2,5-Dihydro-3,4-dimethylfuran tentative	C_6_H_10_O	0.54 ± 0.14^b^	-	-	-	1.06 ± 0.10^a^	6.23	750	732	MS/RI	N	0.98292
VOC.86	85213-22-5	2-Acetyl-1-pyrroline	C_6_H_9_NO	0.15 ± 0.04^a^	0.32 ± 0.11^a^	-	0.15 ± 0.03^a^	0.29 ± 0.14^a^	9.06	945	930	MS/RI	popcorn, toasted, grain, malty	0.89171
VOC.87	143-07-7	Dodecanoic acid	C_12_H_24_O_2_	1.35 ± 0.12^c^	1.76 ± 0.44^bc^	-	2.18 ± 0.27^ab^	-	19.51	1570	1567	MS/RI	mild, fatty, coconut, bay oil	0.94070
VOC.88	3777-69-3	2-Pentylfuran	C_9_H_14_O	1.96 ± 0.16^b^	1.51 ± 0.04^b^	2.56±0.47^a^	1.84 ± 0.04^b^	-	11.02	1004	996	MS/RI	fruity, green, earthy, beany, vegetable, metallic	0.90778
VOC.89	95-48-7	2-Methylphenol	C_7_H_8_O	-	0.19 ± 0.02^a^	-	0.15 ± 0.03^a^	-	12.66	1014	1008	MS/RI	musty, phenolic, plastic, medicinal, herbal, leathery	1.06588
VOC.90	644-30-4	α-Curcumene	C_15_H_22_	-	-	1.18±0.18^a^	-	-	21.08	1524		MS/RI	herbal	0.95965
VOC.91	109-06-8	2-Methylpyridine	C_6_H_7_N	-	-	-	0.05 ± 0.01^a^	-	6.04	787		MS/RI	sweat	1.08581

**Note:** RT is retention time (min), RI^a^ is calculated retention index, RI^b^ is retention index from the NIST Webbook database; N indicates not found in the database, and “-” indicates not found in the sample. Odor descriptions are from http://www.perflavory.com/. Different letters indicate significant differences (*p* < 0.05). All values are means ± SD (n = 3). LYT, NHT, SPT, YLT, and YMT represent *T. eurhizus* from Longyang, Nanhua, Shiping, Yiliang, and Yimen, respectively.

According to origin, there were significant variations in the types and concentrations of VOCs in *T. eurhizus* from different regions (Fig. 3C). Among these, LYT was dominated by esters (27.33 μg/g), which imparted floral, waxy, and fatty aroma characteristics (Li et al., 2024). NHT exhibited a balanced distribution of various compounds, with pyrazines being the most abundant (3.21 μg/g), potentially contributing to its unique baked and nutty aromas. SPT had the highest aldehyde content (17.87 μg/g), contributing fresh grassy, fruity, and fatty aromas (Li et al., 2024). YLT had a higher ketone content than the other origins (16.25 μg/g), and most ketones have low odor thresholds, which may make the fruity, herbal, mushroom, and fatty aromas of YLT more prominent. Furthermore, no hydrocarbons were detected in this origin, and the pyrazine content was the lowest (0.52 μg/g), indicating that its VOCs are concentrated in compounds containing oxygen-bearing functional groups. In contrast, YMT had the highest levels of both alcohols (21.81 μg/g) and hydrocarbons (8.12 μg/g). Alcohols contribute to the characteristic mushroom aroma, while hydrocarbons, despite having higher odor thresholds (Xi et al., 2024), can enhance flavor complexity through synergistic effects, resulting in a prominent mushroom aroma and a richer flavor profile in YMT.

Further multivariate statistical methods were used to distinguish VOCs. The heatmap (Fig. 3D) shows that samples from the same origin cluster well together, while those from different origins are clearly separated, indicating significant regional differences in the accumulation of volatile compounds. Consistent with this, the OPLS-DA model parameters (Fig. 3E) indicate Q^2^ = 0.992, R^2^Y = 0.998, and R^2^X = 0.959, suggesting that the model possesses predictive ability and high explanatory power. Permutation tests (*n* = 200) (Fig. 3F) confirmed the model's reliability, with a Q^2^ intercept of −0.922 (< 0) and an R^2^ intercept of 0.324 (less than R^2^Y = 0.998), indicating that the model has low risk of overfitting and that the results are reliable. The results indicate that samples from the five origins show a clear separation trend on the score plot, with no overlap between groups. YMT clustered in the lower-left quadrant, while SPT clustered on the right. The differences between these two groups were the most significant. Sample points within each group are relatively concentrated, indicating good intra-group reproducibility. These results suggest that the OPLS-DA model can effectively distinguish among the five origins. Meanwhile, based on the threshold of VIP > 1 (Wang, Herrera, et al., 2022), 37 differential marker compounds were identified. Among these, ester compounds accounted for the highest proportion (37.83%), ketone compounds followed, accounting for 24.32%. Notably, 1-octen-3-ol (VIP = 0.941) has a VIP value of less than 1, indicating that the concentration of this compound shows minimal variation across different origins and that it is a characteristic aromatic compound common to *T. eurhizus*.

To summarize, the VOCs of *T. eurhizus* from the five producing areas exhibit significant differences. Esters, aldehydes, ketones, and alcohols constitute the primary components responsible for their aroma characteristics. Specifically, LYT is dominated by esters, NHT is characterized by prominent pyrazines, SPT is rich in aldehydes, YLT shows a concentration of ketones, and YMT features a synergistic enhancement of flavor complexity through alcohols and hydrocarbons, with each producing area displaying a unique aroma profile. The OPLS-DA model effectively distinguished the five producing areas, with esters and ketones serving as key differential markers, while 1-octen-3-ol was determined as a common characteristic aroma component of *T. eurhizus*. These differences in volatile compounds indicate that *T. eurhizus* from different origins can develop region-specific flavor profiles, which supports origin-oriented selection of raw materials for functional food development, such as mushroom seasoning bases, instant soup products, or composite flavor ingredients.

### 3.2 Nutrient content of *T. eurhizus* from different origins

**Fig. 4:**
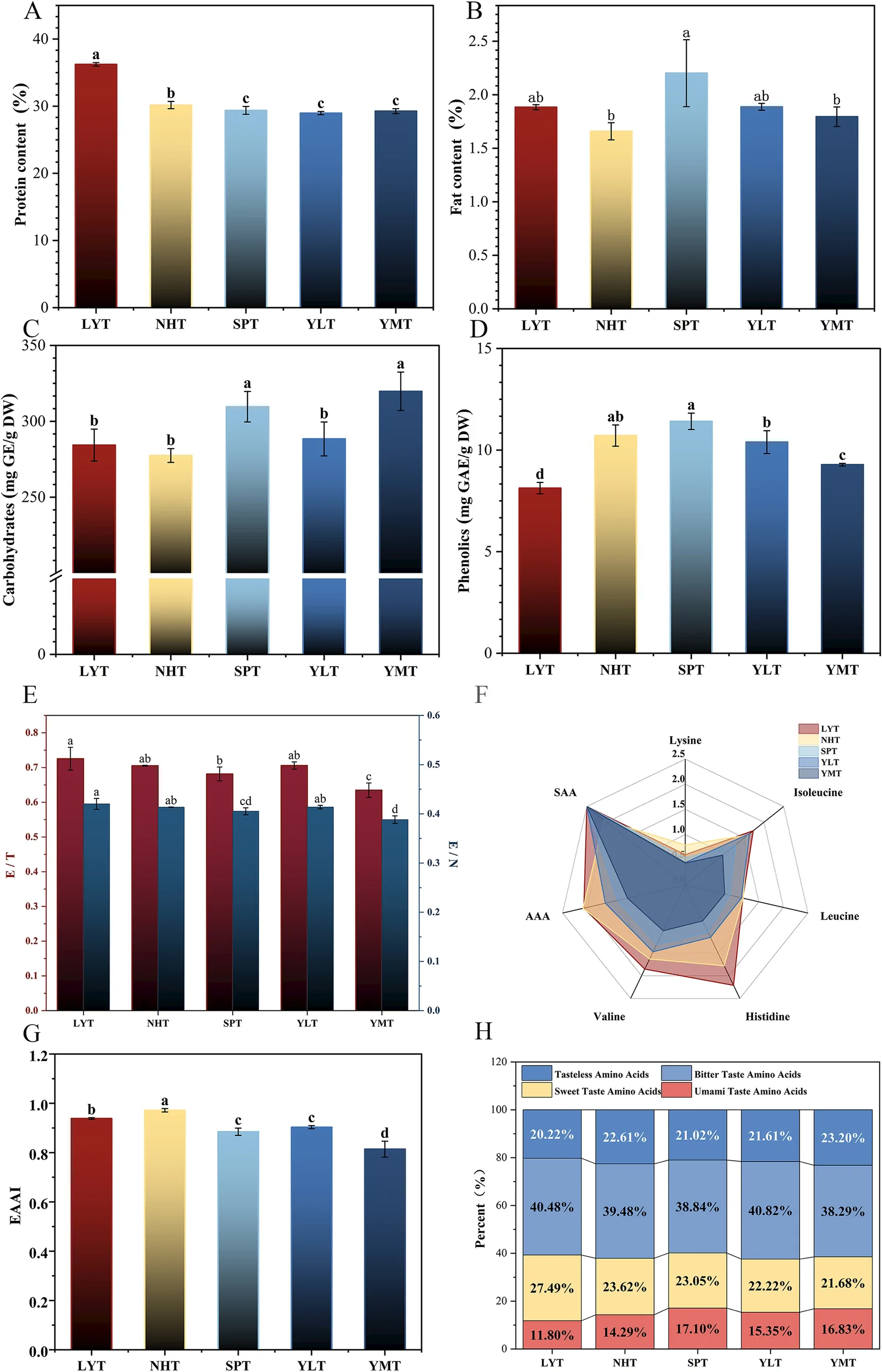
Nutritional composition and amino acid evaluation of *T. eurhizus* from different origins. (A) Protein content, (B) Fat content, (C) Polysaccharide content, (D) Polyphenol content, (E) Ratios of E/T (red) and E/N (blue), (F) Radar chart of AAS, (G) EAAI, and (H) Composition of taste amino acids. SAA refers to sulfur amino acids (methionine + cysteine), and AAA refers to aromatic amino acids (phenylalanine + tyrosine). Different letters indicate significant differences (*p* < 0.05). All values are means ± SD (*n* = 3). LYT, NHT, SPT, YLT, and YMT represent *T. eurhizus* from Longyang, Nanhua, Shiping, Yiliang, and Yimen, respectively. (For interpretation of the references to colour in this figure legend, the reader is referred to the web version of this article.)

Proteins are not only important nutrients but also serve as flavor precursors and play a role in the formation of aromatic compounds. In Fig. 4A, LYT exhibited the highest protein content at 36.22%, followed by NHT at 30.16%. Meanwhile, there was no significant variation in protein content among SPT, YLT, and YMT (*p* > 0.05), with values of 29.37%, 28.70%, and 28.98%, respectively. These are consistent with the protein content range typically reported for edible mushrooms (Boro et al., 2025). This high protein content in LYT may be attributed to the unique ecological environment of this region, which may be more conducive to the absorption and conversion of nitrogen sources by the mycelium, thereby promoting protein synthesis and accumulation. The lack of a significant difference in protein content among the SPT, YLT, and YMT samples suggests that these production areas may share similar environmental characteristics. Fat content is a key indicator for evaluating the nutritional value of food (Hwang et al., 2025). As shown in Fig. 4B, all *T. eurhizus* from different origins exhibited low fat levels (1.66%–2.20%), consistent with the low-fat, health-promoting characteristics of edible mushrooms. Among them, SPT had the highest fat content at 2.20%, which was significantly higher than that of YMT (1.79%) and NHT (1.66%) (*p* < 0.05). In contrast, YLT (1.89%) and LYT (1.88%) exhibited moderate fat content levels, with no significant differences observed compared to the other origins (*p* > 0.05). These results indicate that origin influences fat accumulation in *T. eurhizus*, with the unique ecological environment of the SPT region being more conducive to fat synthesis and accumulation.

Polyphenols and polysaccharides exhibit functions such as regulating blood glucose and lipid levels, providing antioxidant effects, modulating the immune system, and promoting gut health (Cai et al., 2018; Wang et al., 2020). As shown in Fig. 4C, the polysaccharide contents of *T. eurhizus* from different origins were ranked as follows: YMT > SPT > YLT > LYT > NHT. Among these, YMT had the highest polysaccharide content at 319.74 mg GE/g DW, which might be attributed to climatic conditions (e.g., daily temperature fluctuations and precipitation patterns) and soil microbial community structure in its origin that are more conducive to polysaccharide synthesis and accumulation. As shown in Fig. 4D, the polyphenol contents of LYT, NHT, SPT, YLT, and YMT were 8.13, 10.71, 11.42, 10.39, and 9.28 mg GAE/g DW, respectively. Among these, the SPT origin had a significantly higher polyphenol content of 11.42 mg GAE/g DW compared to the other groups. Notably, SPT ranked first in both fat and polyphenol content, indicating a relatively higher energy contribution and stronger antioxidant potential.

Overall, *T. eurhizus* is a food ingredient characterized by high protein, low fat, and richness in polysaccharides and polyphenols. During its development and utilization, differences in the initial levels of bioactive and nutritional constituents among samples from different origins should be considered, as these compounds may exhibit different stability and retention rates during subsequent processing and storage (Afzal et al., 2025; Iqbal et al., 2025).

### 3.3 Amino acid content of *T. eurhizus* from different origins

This experiment identified 17 amino acids, including 7 essential amino acids (EAA) and 10 non-essential amino acids (NEAA). As shown in Table S3, LYT has the highest total EAA content (131.17 mg/g). YMT has the lowest total EAA contents (61.78 mg/g), while NHT, YLT, and SPT have total EAA contents of 107.97, 76.56, and 87.59 mg/g, respectively. The types, amounts, and proportions of these EAAs are key criteria for evaluating the nutritional quality of protein. The ratios of EAA to TAA (E/T) and to NEAA (E/N) are used to evaluate the balance of protein amino acid composition. The FAO/WHO recommended ideal protein has an E/T value of approximately 0.40 and an E/N value of no less than 0.60 (He et al., 2024). As shown in Fig. 4E, the E/N ratios of *T. eurhizus* from the five producing areas range from 0.63 to 0.73, all meeting the ideal pattern of E/*N* ≥ 0.60, indicating a generally balanced amino acid composition across all samples. Among them, LYT exhibits the highest mean E/N value (0.73), reflecting a greater capacity for essential amino acid accumulation in *T. eurhizus* from Longyang. Furthermore, the E/T ratios range from 0.38 to 0.42, all close to the ideal value of 0.40. These results confirm the balanced amino acid composition of *T. eurhizus*. In addition, as shown in the AAS radar plot in Fig. 4F, the lysine score in each of the five samples is at the lowest level among the tested amino acids and exhibits the most pronounced indentation in the radar plot, indicating that lysine is the first limiting amino acid in NHT, LYT, YLT, SPT, and YMT. Among these, NHT shows the highest lysine score (0.80), suggesting a relatively optimal amino acid balance. In contrast, YLT and YMT show lysine scores of 0.45 and 0.44, respectively, and these values are the lowest among all samples, indicating that lysine deficiency is most significant in *T. eurhizus* from these two origins. Further, EAAI was adopted as an evaluation indicator. EAAI reflects how closely the essential amino acid composition of a sample matches the FAO/WHO reference protein. A higher EAAI value indicates higher protein nutritional quality. According to the EAAI grading criteria, an EAAI greater than 0.95 indicates high-quality protein, an EAAI of 0.86–0.95 indicates good protein, an EAAI of 0.75–0.86 indicates usable protein, and an EAAI below 0.75 indicates inadequate protein quality (Sui & Harvey, 2021). As shown in Fig. 4G, the mean EAAI values of the five samples ranked as follows: NHT (0.97) > LYT (0.93) > YLT (0.90) > SPT (0.88) > YMT (0.81). Among them, NHT achieved an EAAI of 0.97, meeting the high-quality protein standard. LYT, YLT, and SPT fell within the good protein range, YMT had the lowest EAAI (0.81), still above 0.75, and thus fell into the usable protein category. These results indicate that the protein nutritional quality varied among samples. NHT exhibited outstanding amino acid balance and protein nutritional quality, making it a high-quality protein resource. It can thus be seen that *T. eurhizus* has a high nutritional profile and that the local environment plays a key role in determining the quality of its protein.

According to their taste properties, amino acids can be categorized into sweet, umami, bitter, and non-taste-bearing amino acids. Fig. 4H illustrates that there are significant differences (*p* < 0.05) in the components of taste-bearing amino acids among *T. eurhizus* from different origins. For umami amino acids, SPT exhibited the highest proportion (17.10%), indicating the strongest umami characteristics among the samples. Furthermore, glutamic acid not only serves as a key contributor to umami taste but also plays an important role in immune regulation (de Aquino et al., 2025). Among all samples, NHT displayed the highest glutamic acid content at 23.90 mg/g. Therefore, NHT may possess potential immune-enhancing benefits. For sweet amino acids, LYT had the highest proportion (27.49%), which was significantly higher than those of other origins (*p* < 0.05), suggesting a potentially sweeter flavor. For bitter amino acids, the proportions across origins were relatively stable. Nevertheless, due to their low gustatory intensity, bitter amino acids are typically masked by sweet amino acids, soluble sugars, or other flavor compounds, and thus are not readily perceived as bitter by consumers (Sun et al., 2020).

In summary, *T. eurhizus* has a complete range of amino acids with a balanced composition that conforms to the FAO/WHO ideal protein pattern. Among all samples, NHT exhibited the optimal nutritional quality. Moreover, the growing environment exerts a significant regulatory effect on amino acid composition, taste characteristics, and protein nutritional value.

### 3.4 5′-Nucleotide content in *T. eurhizus* from different origins

The 5′-nucleotides are critical components contributing to the umami flavor of edible mushrooms (Sun et al., 2020). In this study, five 5′-nucleotides were identified, including 5′-AMP, 5′-IMP, 5′-GMP, 5′-CMP, and 5′-UMP. As shown in Table 2, the total 5′-nucleotide contents of LYT, NHT, SPT, YLT, and YMT were 26.67, 13.53, 13.36, 13.84, and 18.73 mg/g, respectively. Among these, the total nucleotide content of LYT was markedly higher than those of the other origins (*p* < 0.05). From the nucleotide composition, 5′-CMP and 5′-GMP were the major components, accounting for 26.70%–46.36% and 23.80%–30.37% of the total nucleotide content, respectively. From a umami perspective, 5′-GMP is the nucleotide with the strongest ability to impart umami flavor in edible mushrooms (Zhou et al., 2025). Not only does it induce flavor at extremely low concentrations, but it also produces a strong synergistic effect with glutamic acid (Gao et al., 2021). 5′-AMP itself has a sweet taste and can effectively suppress bitterness. Meanwhile, 5′-IMP is the primary source of umami in meat. Among key flavor-contributing nucleotides, LYT stood out, with 5′-GMP, 5′-AMP, and 5′-IMP contents exceeding those of the other origins, at 8.20, 2.65, and 0.56 mg/g, respectively. Therefore, LYT possesses potential advantages in terms of umami, sweetness, and flavor complexity. Conversely, 5′-CMP and 5′-UMP have lower umami activity and make a limited contribution to umami (Tanaka et al., 2025), but differences in their contents may reflect variations in the nucleotide metabolic pathways among *T. eurhizus* from different origins. Further TAV analysis (Table 3) showed that the TAV values for 5′-AMP and 5′-GMP from all origins were significantly greater than 1. This indicates that the flavor profile of nucleotides in *T. eurhizus* is primarily composed of a synergistic combination of the umami, sweetness, and richness contributed by 5′-GMP and 5′-AMP. Notably, 5′-AMP imparts a sweet taste and effectively suppresses bitterness. Combined with amino acid content analysis, this may also be a key reason why *T. eurhizus*, despite containing bitter amino acids, does not exhibit a pronounced bitter or astringent sensation. These results agree with the conclusions presented by Hu et al. (2020) in their study of *S. rugoso-*annulata. In summary, LYT possesses higher total 5′-nucleotide and key taste-determining nucleotide (5′-GMP, 5′-AMP) contents, as well as richer umami, sweetness, and body, making it an excellent material for studying umami enhancement and flavor quality regulation in the processing of *T. eurhizus*.

**Table 2:** 5′-Nucleotides and soluble sugars content of *T. eurhizus* from different regions.

Compounds		Content (mg/gdryweight)				
		LYT	NHT	SPT	YLT	YMT
5′-Nucleotides	5′-CMP	12.15 ± 0.14^a^	5.36 ± 0.36^d^	3.61 ± 0.15^e^	5.69 ± 0.02^c^	8.68 ± 0.05^b^
	5′-UMP	3.45 ± 0.01^a^	2.46 ± 0.04^c^	3.42 ± 0.02^a^	2.39 ± 0.02^d^	2.76 ± 0.003^b^
	5′-GMP	8.20 ± 0.88^a^	3.22 ± 0.000017^d^	4.12 ± 0.03^b^	4.13 ± 0.02^b^	4.93 ± 0.02^c^
	5′-IMP	0.56 ± 0.003^a^	0.54 ± 0.01^a^	0.48 ± 0.01^b^	0.48 ± 0.02^b^	0.41 ± 0.03^c^
	5′-AMP	2.65 ± 0.04^a^	1.95 ± 0.03^b^	1.73 ± 0.10^c^	1.16 ± 0.02^d^	1.95 ± 0.04^b^
	Total	26.67 ± 0.71^a^	13.53 ± 0.34^c^	13.36 ± 0.19^c^	13.84 ± 0.02^c^	18.73 ± 0.08^b^
Soluble sugars	Trehalose	58.89 ± 0.20^e^	115.09 ± 0.28^a^	89.13 ± 1.18^c^	62.64 ± 0.20^d^	97.38 ± 0.98^b^
	Fucose	0.23 ± 0.004^b^	0.47 ± 0.03^a^	0.42 ± 0.03^a^	0.42 ± 0.04^a^	0.29 ± 0.02^b^
	Rhamnose	0.10 ± 0.04^c^	1.25 ± 0.01^a^	1.29 ± 0.02^a^	0.82 ± 0.08^b^	0.86 ± 0.01^b^
	Galactose	80.60 ± 0.01^e^	154.70 ± 0.06^d^	181.76 ± 1.22^b^	166.15 ± 0.84^c^	192.31 ± 0.13^a^
	Glucose	1.25 ± 0.05^d^	3.98 ± 0.07^b^	3.57 ± 0.12^c^	3.61 ± 0.11^c^	4.42 ± 0.11^a^
	Total	141.07 ± 0.05^d^	275.49 ± 0.10^b^	276.17 ± 4.97^b^	233.64 ± 0.80^c^	295.26 ± 1.05^a^

**Note:** Different letters indicate significant differences (*p* < 0.05). All values are means ± SD (*n* = 3). LYT, NHT, SPT, YLT, and YMT represent *T. eurhizus* from Longyang, Nanhua, Shiping, Yiliang, and Yimen, respectively.

**Table 3:** Non-volatile organic compounds with TAV > 1 in T. *eurhizus* from different origins.

Compounds	TTV (mg/g)	TAV				
		LYTM	NHTM	SPTM	YLTM	YMTM
Trehalose	10.61	5.55	10.85	8.40	5.90	9.18
Glucose	3.24	24.88	47.75	56.10	51.28	59.36
5′-GMP	0.26	32.18	12.63	16.17	16.19	19.33
5′-AMP	0.13	21.17	15.61	13.86	9.29	15.58

**Note:** LYT, NHT, SPT, YLT, and YMT represent *T. eurhizus* from Longyang, Nanhua, Shiping, Yiliang, and Yimen, respectively.

### 3.5 Soluble sugars content in *T. eurhizus* from different origins

Soluble sugars are the principal compounds associated with the sweetness and texture of edible mushrooms (Hu et al., 2020). Table 2 shows that the soluble sugar contents of YMT, LYT, NHT, SPT, and YLT were 295.26, 141.07, 275.49, 276.17, and 233.64 mg/g, respectively. Of these, YMT had the highest soluble sugar content, indicating that it is richest in flavor precursors. The composition revealed that glucose and trehalose were the primary soluble sugar components, accounting for 56.15%–71.11% and 26.81%–41.77% of the total soluble sugars, respectively. Among these, glucose is a direct sweetening agent and also the core substrate for the Maillard reaction. YMT (192.31 mg/g) had the highest glucose content, followed by SPT (181.76 mg/g) and YLT (166.15 mg/g), with NHT (154.70 mg/g) in the middle and LYT (80.60 mg/g) having the lowest. This indicates that YMT and SPT have a higher potential for contributing sweetness and a stronger potential for the Maillard reaction. Trehalose, as a characteristic stress metabolite of mushrooms, accumulates under adverse conditions such as drought and low temperatures (Eh et al., 2024). The trehalose contents in NHT (115.09 mg/g) and YMT (97.38 mg/g) were markedly higher than those of the other origins, which may be attributed to specific environmental conditions in Nanhua and Yimen (such as temperature, humidity, altitude, and soil characteristics) that favor the synthesis and accumulation of trehalose. Furthermore, according to Table 3, the TAV values for both glucose and trehalose were greater than 1 in all samples, indicating that these two sugars are the primary contributors to the sweetness and texture of *T. eurhizus*. In summary, the soluble sugar content of *T. eurhizus* exhibits origin-specificity, and its sweetness primarily derives from glucose and trehalose.

The differences in non-volatile compounds further indicate that *T. eurhizus* from different origins has distinct roles in nutritional attributes and taste potential. In particular, the LYT showed higher protein and high-quality amino acid levels, making it more suitable as a nutrition-enhancing raw material; the LYT contained higher levels of 5′-nucleotides, especially favoring the development of umami-enhancing products; and the SPT and YMT exhibited greater advantages in polysaccharides and soluble sugars, making them more promising for flavor-improving or functional ingredient applications. Meanwhile, the coordinated changes among amino acids, nucleotides, and sugars suggest that the quality of *T. eurhizus* is not determined by a single component, but by the combined action of multiple non-volatile substances, providing a scientific basis for targeted utilization, blending, and further processing. This multi-component synergistic pattern of quality formation has also been demonstrated in functional studies of grape seed extract (Sajid et al., 2025).

### 3.6 Analysis of the correlation between volatile and non-volatile organic compounds in *T. eurhizus*

**Fig. 5:**
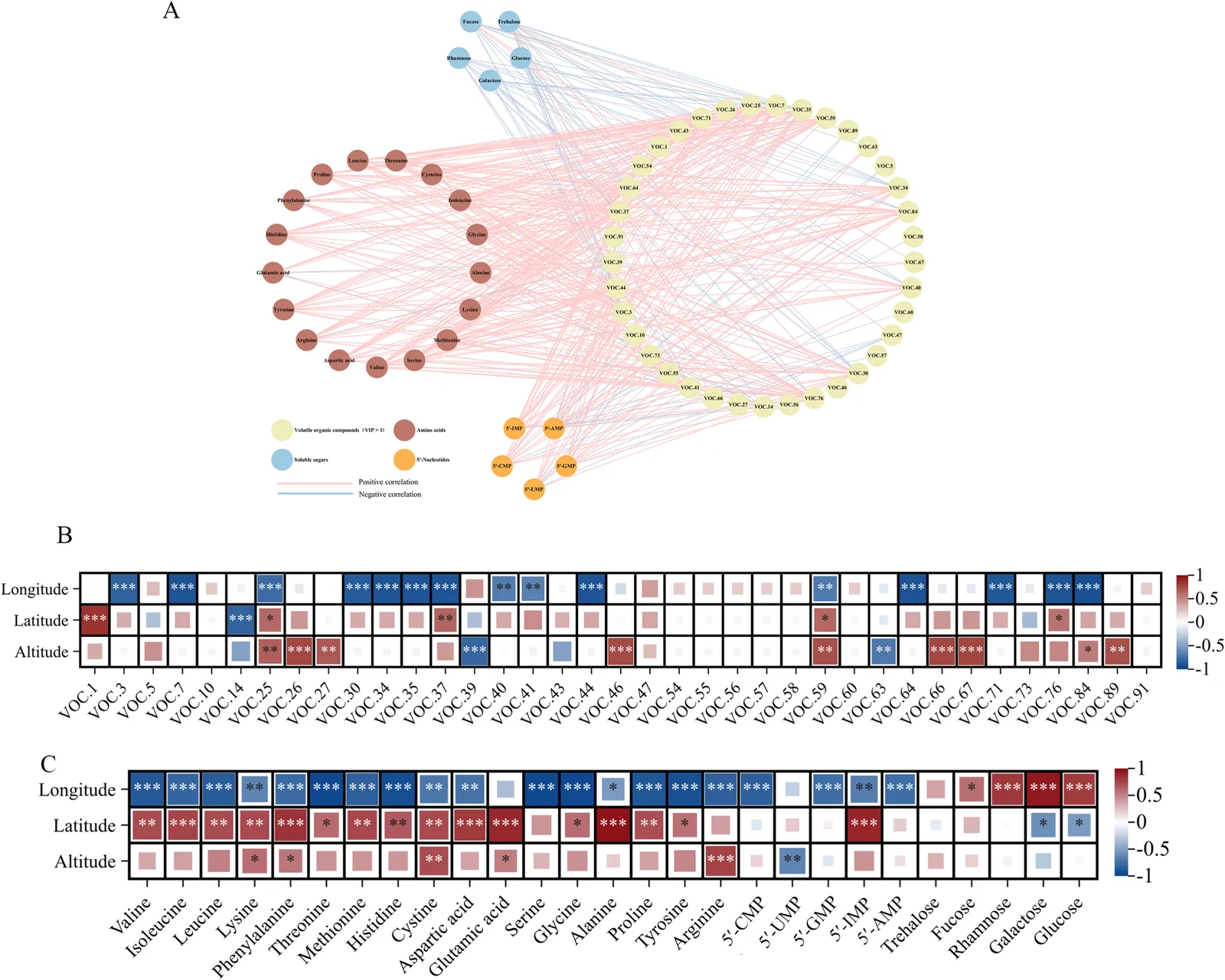
Pearson correlation network analysis of metabolites in *T. eurhizus* from five origins. (A) Correlation network of 5′-nucleotides, soluble sugars, amino acids, and volatile organic compounds (VIP > 1). Only correlations with |r| > 0.6 are displayed. (B) Pearson correlations between environmental factors and volatile organic compounds (VIP > 1). (C) Pearson correlations between environmental factors and non-volatile organic compounds. Red and blue represent positive and negative correlations, respectively. (For interpretation of the references to colour in this figure legend, the reader is referred to the web version of this article.)

This research employed Pearson correlation analysis to elucidate the interrelationships among 5′-nucleotides, soluble sugars, amino acids, and VOCs with VIP > 1 (Fig. 5A). The results show that amino acids exhibit strong positive correlations with numerous VOCs. For example, serine, glycine, and threonine show strong positive correlations (*r* = 0.88–0.98) with Benzyl alcohol (VOCs.3), Ethyl 4-oxovalerate (VOCs.34), and Methyl phenylacetate (VOCs.37). This is because amino acids can be converted into aldehydes, alcohols, and heterocyclic compounds through metabolic pathways such as Strecker degradation, decarboxylation, or deamination, thereby contributing to the formation of VOCs (Liu et al., 2023). Meanwhile, Wang et al. (2023) similarly stated that amino acids can generate volatile alcohols and aldehydes through enzymatic degradation. Moreover, 5′-CMP and 5′-GMP exhibited strong positive correlations (*r* = 0.84–0.95) with Benzyl alcohol (VOCs.3), Isophytol (VOCs.7), Ethyl lactate (VOCs.30), Ethyl 4-oxovalerate (VOCs.34), and Ethyl heptanoate (VOCs.35). This is because 5′-nucleotides are broken down into smaller molecules by enzymes (such as proteases and nucleases), and these small molecules can act as building blocks for creating other flavor compounds (Huang et al., 2019; Zhou et al., 2025). Conversely, soluble sugars exhibit negative correlations with esters and hydrocarbons. For example, galactose correlates negatively with Ethyl 4-oxovalerate (VOCs.34, *r* = −0.95) and Di-epi-α-cedrene (VOCs.76, *r* = −0.92). This difference may stem from their distinct metabolic roles: during the phase of intensive VOC synthesis, soluble sugars are primarily consumed as energy sources and carbon skeletons (Sun et al., 2024), rather than being directly converted into specific VOCs. Overall, the correlation analysis confirms that VOCs and NVOCs synergistically shape the unique quality of *T. eurhizus* mushrooms.

### 3.7 Correlation analysis of volatile and non-volatile compounds in *T. eurhizus* in relation to environmental factors

Environmental factors, including longitude, latitude, and altitude, influence the synthesis of metabolites in mushrooms (Zhou et al., 2025). This study analyzed the correlations between NVOCs (17 amino acids, 7 nucleotides, and 4 soluble sugars) and VOCs with VIP > 1 in *T. eurhizus* and the latitude, altitude, and longitude of the sampling sites. The *r* values are shown in Fig. 5B and Fig. 5C. Longitude was negatively correlated with amino acids, 5′-nucleotides, esters, and hydrocarbons. For example, the *r* values between longitude and serine, glycine, threonine, 5′-CMP, ethyl 4-oxovalerate (VOCs.34), and Di-epi-α-cedrene (VOCs.76) ranged from −0.84 to −0.96. Conversely, longitude exhibits strong positive correlations with soluble sugars (*r* = 0.94 for galactose and *r* = 0.81 for glucose). This indicates that as longitude increases, the contents of 5′-nucleotides, amino acids, esters, and hydrocarbons decrease, while the contents of soluble sugars increase. Latitude was positively correlated with amino acids, nucleotides, aldehydes, and hydrocarbons. For example, the *r* values between latitude and phenylalanine, cysteine, 5′-IMP, 1-Hexanol (VOCs.1), and Di-epi-α-cedrene (VOCs.76) ranged from 0.61 to 0.97, indicating that as latitude increases, the concentrations of amino acids, 5′-nucleotides, aldehydes, and hydrocarbons tend to rise. Research by Andersen et al. (2022) also indicates that latitude and longitude are associated with flavor compounds.

In addition, altitude may indirectly regulate microbial community structure and function by influencing soil physicochemical properties (such as organic matter, total organic carbon, and key nutrients) (Siles & Margesin, 2016), thereby affecting the synthesis of flavor compounds. Altitude is positively correlated with most NVOCs, with amino acids showing the most significant response. For example, altitude is strongly positively correlated with arginine (*r* = 0.82). Moreover, altitude shows strong positive correlations with 2-Pinen-4-one (VOCs.66), 2-Methyl-3-hexanone (VOCs.67), 2-Ethylacrolein (VOCs.26), and Dodecanal (VOCs.27) among others (*r* = 0.71–0.79). This variation is likely caused by strong ultraviolet radiation at high altitudes, which activates the propanediol metabolic cascade and promotes the formation of flavor compounds (Salomé-Abarca et al., 2020). In summary, longitude, latitude, and altitude all contribute to the synthesis and accumulation of VOCs and NVOCs in *T. eurhizus.* Similar phenomenon has also been reported in other natural food materials, where chemical profiles are influenced by geographic origin and growing conditions (Khawaja et al., 2025). Nevertheless, the associations between geocoordinates and VOCs/NVOCs require further study.

## 4 Conclusion

This study compared differences in VOCs and NVOCs among *T. eurhizus* from five regions in Yunnan Province (Yiliang, Nanhua, Yimen, Longyang, and Shiping), revealing the influence of origin on their quality. The results showed that, for VOCs, a total of 91 compounds were identified, with esters, aldehydes, ketones, and alcohols accounting for 78.02% of the total. The OPLS-DA model showed discriminant capacity among the five origins, identifying esters and ketones as potential regional markers, while 1-octen-3-ol was a common characteristic aroma component across all samples. For NVOCs, the analysis revealed a generally balanced amino acid composition across the five samples. LYT exhibited the highest proportion of sweet amino acids (27.49%) and the highest total 5′-nucleotides (26.67 mg/g); NHT demonstrated the best protein quality (EAAI = 0.97); SPT was richest in umami amino acids (17.10%) and polyphenols (11.42 mg/g); YMT contained the highest levels of polysaccharides (319.74 mg/g) and soluble sugars (295.26 mg/g). Notably, all samples had TAV > 1 for 5′-GMP, 5′-AMP, glucose, and trehalose, confirming their key roles in umami and sweetness. Correlation analysis indicated that there was a close metabolic association between VOCs and NVOCs, and that environmental factors exerted varying degrees of influence on metabolite accumulation.

To summarize, origin is a critical factor shaping flavor and nutritional quality of *T. eurhizus*, with different origins exhibiting distinct advantages in terms of flavor characteristics and nutritional value. This study offers a scientific foundation for traceability, quality evaluation, development of specialty products, and targeted processing of *T. eurhizus,* and is important for improving its industrial value. Although *T. eurhizus* has a long history of consumption in Yunnan and is generally considered safe, future industrial development should still integrate raw-material traceability, quality control, and assessment of potential contamination risks to improve its safety and credibility as a functional food ingredient (Nadeem et al., 2025). Notably, the quantitative analysis of key environmental variables—including soil nutrient cycling, microbial community dynamics, and climatic fluctuations—has not yet been conducted. Future studies should also investigate whether different termite species or colony-level factors influence metabolite accumulation in *T. eurhizus*. On this basis, metabolomics, metagenomics, and other multi-omics approaches could be employed to elucidate the microbial-driven biosynthetic pathways of flavor compounds, thereby providing a more comprehensive understanding of the “environment-microbe-flavor” regulatory mechanism.

## CRediT authorship contribution statement

**Qian Zhang:** Writing – original draft, Formal analysis, Data curation, Conceptualization. **Yanling Lu:** Formal analysis, Data curation, Conceptualization. **Ying Chen:** Formal analysis, Data curation. **Fuyan Li:** Formal analysis, Data curation. **Jiaojiao Yang:** Resources, Project administration, Funding acquisition, Conceptualization. **Long Han:** Project administration, Funding acquisition, Conceptualization. **Qian Ma:** Writing – review & editing, Validation, Supervision. **Fangyu Fan:** Writing – review & editing, Supervision, Resources, Project administration, Funding acquisition, Conceptualization.

## Declaration of competing interest

The authors declare that they have no known competing financial interests or personal relationships that could have appeared to influence the work reported in this paper.

## Data Availability

Data will be made available on request.

## Appendix A Supplementary data

Supplementary material Tables S1–S4 provide sample collection information, electronic-nose sensor analytes, amino acid contents, and representative chemical compounds
