# Supplementary material for: Volatile and non-volatile organic compounds synergistically determine quality in Termitomyces eurhizus from different regions
Source: Food Chem X. 2026 Aug 13;38:104320. doi: 10.1016/j.fochx.2026.104320 (PMC13519606; doi:10.1016/j.fochx.2026.104320)
Supplement: Supplementary file 1 [file mmc1.docx]

**Volatile and Non-volatile Organic Compounds Synergistically Determine Quality in *Termitomyces eurhizus* from Different Regions**

**Qian Zhang ^a, b^, Yanling Lu ^a, b^, Ying Chen ^a, b^, Fuyan Li ^a, b^, Jiaojiao Yang ^a, b^, Long Han ^a, b^, Qian Ma ^a, b,^ *, Fangyu Fan ^c,^ ***

^a^ College of Biological Science and Food Engineering, Southwest Forestry University, Kunming 650224, China

^b^ Forest Resources Exploitation and Utilization Engineering Research Center for Grand Health of Yunnan Provincial Universities, Southwest Forestry University, Kunming 650224, China

^c^ Key Laboratory of Forest Disaster Warning and Control of Yunnan Province, Southwest Forestry University, Kunming 650224, China

*** Corresponding author.** College of Biological Science and Food Engineering, Southwest Forestry University, Kunming 650224, China.

E-mail addresses: maqian0224@163.com (Q. Ma), ffy118@163.com (F. Fan).

**Table S1**

Collection Information of *Termitomyces eurhizus* from Different Origins.

| Collected Location | Longitude | Latitude | Altitude | Climate |
| --- | --- | --- | --- | --- |
| Longyang District, Baoshan City, Yunnan Province | 99°9′44″ | 25°6′51″ | 1650 | Subtropical plateau monsoon climate |
| Nanhua County, Chuxiong Yi Autonomous Prefecture, Yunnan Province | 101°16′26″ | 25°11′31″ | 1857 | Subtropical plateau monsoon climate |
| Shiping County, Honghe Hani and Yi Autonomous Prefecture, Yunnan Province | 102°29′38″ | 23°42′22″ | 1413 | Subtropical plateau monsoon climate |
| Yiliang County, Kunming City, Yunnan Province | 103°08′30″ | 24°55′12″ | 1650 | Subtropical plateau monsoon climate |
| Yimen County, Yuxi City, Yunnan Province | 102°09′47″ | 24°40′19″ | 1601 | Subtropical plateau monsoon climate |

| **Table S2**  Target Analytes for E-Nose Sensors | |
| --- | --- |
| **Sensors** | **Sensitivity** |
| W1C | Aromatics, benzene |
| W3C | Ammonia, aromatics |
| W5C | Alkane, aromatics |
| W1S | Short-chain alkanes |
| W2S | Alcohols, ethers, aldehydes, ketones |
| W3S | Long-chain alkanes |
| W5S | Nitrogen oxides |
| W6S | Hydrogen |
| W1W | Inorganic sulfides |
| W2W | Aromatics, organic sulfides |

**Table S3**

Amino acid content of *T. eurhizus* from different regions

|  | | Content (mg/g dry weight) | | | |  |
| --- | --- | --- | --- | --- | --- | --- |
|  |  | LYT | NHT | SPT | YLT | YMT |
| Essential Amino Acids (EAA) | Valine | 23.48 ± 0.62^a^ | 17.23 ± 0.46^b^ | 13.49 ± 1.22^d^ | 14.93 ± 0.93^c^ | 10.32 ± 0.69^e^ |
|  | Isoleucine | 17.50 ± 0.63^a^ | 13.41 ± 0.63^b^ | 10.47 ± 0.58^c^ | 13.24 ± 0.16^b^ | 7.78 ± 0.43^d^ |
|  | Leucine | 28.04 ± 0.69^a^ | 23.50 ± 0.64^b^ | 17.59 ± 0.54^d^ | 21.90 ± 0.33^c^ | 15.51 ± 0.68^e^ |
|  | Lysine | 12.63 ± 0.36^b^ | 13.91 ± 0.79^a^ | 9.14 ± 0.93^c^ | 7.50 ± 0.33^d^ | 7.40 ± 0.24^d^ |
|  | Threonine | 19.78 ± 0.71^a^ | 13.19 ± 0.32^b^ | 7.78 ± 0.42^d^ | 9.96 ± 0.38^c^ | 7.46 ± 0.61^d^ |
|  | Phenylalanine | 24.31 ± 0.53^a^ | 22.46 ± 1.16^b^ | 15.03 ± 0.88^c^ | 16.51 ± 1.73^c^ | 10.99 ± 0.80^d^ |
|  | Methionine | 5.44 ± 0.45^a^ | 4.28 ± 0.30^b^ | 3.07 ± 0.46^c^ | 3.56 ± 0.20^c^ | 2.31 ± 0.31^d^ |
|  | Total EAA | 131.17 ± 3.28^a^ | 107.97 ± 2.28^b^ | 76.56 ± 4.00^d^ | 87.59 ± 3.04^c^ | 61.78 ± 3.15^e^ |
| Non-essential Amino Acids (NEAA) | Histidine | 15.21 ± 0.44^a^ | 10.20 ± 0.15^b^ | 5.81 ± 0.42^c^ | 6.36 ± 0.30^c^ | 4.40 ± 0.48^d^ |
|  | Tyrosine | 22.80 ± 3.85^a^ | 17.09 ± 0.19^b^ | 10.48 ± 0.50^c^ | 13.17 ± 0.78^c^ | 10.69 ± 0.46^c^ |
|  | Cystine | 27.70 ± 0.64^a^ | 28.02 ± 0.64^a^ | 20.07 ± 1.05^c^ | 25.07 ± 1.23^b^ | 18.81 ± 0.60^c^ |
|  | Aspartic acid | 15.34 ± 0.86^a^ | 13.40 ± 0.53^b^ | 11.48 ± 1.45^c^ | 12.41 ± 0.35^bc^ | 9.19 ± 0.52^d^ |
| *(continued on next page)* | | | | | | |
| **Table S3 (continued)** | | | | | | |
| **Amino Acids** | | Content (mg/g dry weight) | | | |  |
|  |  | LYT | NHT | SPT | YLT | YMT |
| Non-essential Amino Acids (NEAA) | Glutamic acid | 21.50 ± 0.65^bc^ | 23.90 ± 0.94^a^ | 20.81 ± 0.18^c^ | 20.09 ± 0.65^c^ | 17.58 ± 0.79^d^ |
|  | Serine | 12.79 ± 0.74^a^ | 7.42 ± 0.25^b^ | 4.91 ± 0.39^c^ | 5.09 ± 0.76^c^ | 5.65 ± 0.32^c^ |
|  | Glycine | 18.44 ± 0.30^a^ | 11.62 ± 0.25^b^ | 5.17 ± 0.84^cd^ | 6.08 ± 0.35^c^ | 4.91 ± 0.91^d^ |
|  | Alanine | 22.06 ± 0.74^a^ | 20.15 ± 0.69^b^ | 18.82 ± 0.27^c^ | 17.76 ± 0.79^d^ | 11.14 ± 0.19^e^ |
|  | Proline | 12.75 ± 0.47^a^ | 9.29 ± 0.27^b^ | 6.84 ± 0.65^d^ | 8.16 ± 0.39^c^ | 5.30 ± 0.19^e^ |
|  | Arginine | 12.39 ± 0.44^a^ | 12.02 ± 0.63^a^ | 7.89 ± 0.97^c^ | 9.93 ± 0.63^b^ | 9.59 ± 0.86^b^ |
|  | Total NEAA | 180.99 ± 6.13^a^ | 153.11 ± 3.51^b^ | 112.28 ± 3.82^d^ | 124.11 ± 3.23^c^ | 97.28 ± 2.26^e^ |

**Note:** Different letters indicate significant differences (*p* < 0.05). All values are means ± SD (n = 3). LYT, NHT, SPT, YLT, and YMT represent *T. eurhizus* from Longyang, Nanhua, Shiping, Yiliang, and Yimen, respectively.

**Table S4**

Representative chemical compounds studied in this work

| No. | Chemical compound name | PubChem CID | Type |
| --- | --- | --- | --- |
| 1 | 1-Octen-3-ol | [18827](https://pubchem.ncbi.nlm.nih.gov/compound/18827) | Volatile compound |
| 2 | (E)-2-Nonenal | [5283335](https://pubchem.ncbi.nlm.nih.gov/compound/5283335) | Volatile compound |
| 3 | Methyl hexanoate | [7824](https://pubchem.ncbi.nlm.nih.gov/compound/7824) | Volatile compound |
| 4 | Guanosine 5'-monophosphate | [135398631](https://pubchem.ncbi.nlm.nih.gov/compound/135398631) | Non-volatile compound |
| 5 | Adenosine 5'-monophosphate | [6083](https://pubchem.ncbi.nlm.nih.gov/compound/6083) | Non-volatile compound |
| 6 | Glucose | [5793](https://pubchem.ncbi.nlm.nih.gov/compound/5793) | Non-volatile compound |
| 7 | Trehalose | [7427](https://pubchem.ncbi.nlm.nih.gov/compound/7427) | Non-volatile compound |
